# The Short-Term Course of Nonsuicidal Self-Injury Among Individuals Seeking Psychiatric Treatment

**DOI:** 10.1001/jamanetworkopen.2024.40510

**Published:** 2024-10-22

**Authors:** Glenn Kiekens, Laurence Claes, Evan M. Kleiman, Koen Luyckx, Daniel D. L. Coppersmith, Rebecca G. Fortgang, Inez Myin-Germeys, Matthew K. Nock

**Affiliations:** 1 Department of Medical and Clinical Psychology, Tilburg University, Tilburg, the Netherlands; 2 Faculty of Psychology and Educational Sciences, Clinical Psychology, KU Leuven, Leuven, Belgium; 3 Department of Neurosciences, Center for Contextual Psychiatry, KU Leuven, Leuven, Belgium; 4 Faculty of Medicine and Health Sciences, University of Antwerp, Antwerp, Belgium; 5 Department of Psychology, Rutgers, State University of New Jersey, Piscataway; 6 Unit for Professional Training and Service in the Behavioural Sciences, University of the Free State, Bloemfontein, South Africa; 7 Department of Psychology, Harvard University, Cambridge, Massachusetts; 8 Department of Psychiatry, Massachusetts General Hospital, Boston

## Abstract

**Importance:**

A major portion of adolescents and adults seeking psychiatric treatment report nonsuicidal self-injury (NSSI) within the past month, yet the short-term course of NSSI among these patients remains poorly understood.

**Objective:**

To advance the understanding of the short-term course of NSSI cognitions (ie, thoughts, urges, and self-efficacy to resist self-injury) and behavior.

**Design, Setting, and Participants:**

A cohort study was conducted using an intensive longitudinal design with ecological momentary assessment, including 6 daily surveys and event registrations of self-injury for 28 days. Data were collected from June 2021 to August 2023. Individuals using mental health services in the Flanders region in Belgium reporting past-month NSSI urges and/or behavior at intake were recruited by referral.

**Main Outcomes and Measures:**

Nonsuicidal self-injury thoughts, urges, self-efficacy, and behavior. Sociodemographic and clinical baseline characteristics served as between-person variables. Descriptive and variability statistics and dynamic structural equation modeling were used.

**Results:**

Participants completed a mean (SD) of 121 (34.5) surveys, totaling 15 098 longitudinal assessments (median adherence, 78.6%; IQR, 59.5%-88.7%). Among 125 patients (87.2% female; median age, 22.0 [range, 15-39] years; 52.8% heterosexual), NSSI thoughts and urges were present during most assessments but were low in intensity (individual means [SD] on a 0- to 6-point scale, 1.52 [1.13] for urges; 1.57 [1.18] for thoughts). The prevalence of NSSI behavior was 84.0% monthly, 49.90% weekly, and 18.19% daily. Between-patient variability was substantial (intraclass correlation coefficient, 0.43-0.47; range of individual means for cognitions, 0-6; individual frequency behavior, 0-103), with recency and frequency of NSSI thoughts and behavior at intake consistently associated with individual differences in the course of NSSI. The greatest variability was observed within patients (root mean square of successive differences from 1.31 for self-efficacy to 1.40 for instability of thoughts), characterized by changes in the intensity of cognitions by more than 1 within-person SD between assessments less than 2 hours apart in 1 of 5 instances. Nonsuicidal self-injury behavior is rare in the morning, increased in the afternoon, and most frequent in the evening. Nonsuicidal self-injury cognitions were contemporaneous and temporally associated with each other’s course, with higher-than-usual thoughts and lower self-efficacy uniquely signaling heightened risk for NSSI behavior in the next 2 hours.

**Conclusions and Relevance:**

In this cohort study of treatment-seeking individuals, NSSI cognitions and behavior appeared to be dynamic over the short term. These findings suggest the potential utility of self-monitoring outside the therapy setting and the need to focus assessment and interventions on the evening hours.

## Key Points

**Question:**

What is the short-term course of nonsuicidal self-injury (NSSI) among individuals seeking psychiatric treatment?

**Findings:**

In this cohort study, 125 treatment-seeking adolescents and adults who self-injure responded to 15 098 ecological momentary assessments of NSSI cognitions and behavior and registered self-injury. The course of NSSI was heterogeneous and highly dynamic, with self-injury being rare in the morning, increased in the afternoon, and most frequent in the evening.

**Meaning:**

These findings suggest that real-time interventions are needed for individuals who self-injure, particularly in the evening hours, and they highlight the importance of ecological momentary assessments for assessing risk moments outside the therapy room.

## Teaser

This cohort study examines the association of nonsuicidal self-injury (NSSI) cognition and behavior, reported using ecological momentary assessment, with subsequent NSSI.

## Introduction

Nonsuicidal self-injury (NSSI), the direct and deliberate damage of one’s body tissue without suicidal intent (eg, cutting or hitting oneself), is a major mental health concern worldwide. Approximately 1 in 5 individuals have an onset of NSSI before age 25 years,^1,2^ with half of adolescents and 1 in 10 adults (especially females) who receive psychiatric treatment reporting past-month NSSI.^3,4,5,6^ Nonsuicidal self-injury is uniquely associated with an increased risk for rehospitalization and suicide attempts.^7,8,9,10^ These findings underscore the clinical importance of NSSI, a condition formally recognized by the American Psychiatric Association in the *Diagnostic and Statistical Manual of Mental Disorders, Fifth Edition, Text Revision* (*DSM-5-TR*).^11^ Despite progress in understanding the prevalence, correlates, and longer-term course of NSSI among individuals seeking treatment,^12,13^ its course over hours, days, and weeks during treatment remains poorly understood. As a result, it remains challenging to intervene effectively.^14^

The lack of progress in understanding the short-term course can be attributed in part to the absence of methods that allow direct observation of NSSI thoughts (ie, thinking about deliberately hurting oneself), NSSI urges (ie, a difficult-to-control desire to self-injure), perceived self-efficacy to resist NSSI (ie, belief in one’s ability not to engage in self-injury), and NSSI behavior (ie, engagement in self-injury).^15,16,17^Advances in digital technology and the use of ecological momentary assessment (EMA), also called experience sampling method,^18,19^ enable real-time tracking of NSSI cognitions and behavior.^20^ Initial findings show that EMA outperforms retrospective interviews in identifying episodes of suicidal thinking and patients who engage in NSSI.^21,22^ While EMA studies have focused on individuals in the community setting^15,23,24,25,26,27^ and patients with borderline personality disorder (BPD),^28,29,30^ information on the naturalistic course of NSSI is lacking among individuals seeking mental health care.

To provide a reference point, this study presents empirical data from the Detection of Acute Risk of Self-Injury project.^17^ The objectives were to clarify the short-term course of NSSI and investigate whether the pattern of cognitions and behavior (1) varies between treatment-seeking individuals, (2) varies within treatment-seeking individuals, and (3) covaries and can be estimated across hours. In addition, the study evaluated whether sociodemographic and clinical characteristics are associated with differences in the course of NSSI during treatment between individuals.

## Methods

### Design and Participants

This cohort study used EMA among patients with past-month NSSI receiving psychiatric treatment between June 2021 and August 2023. The study adheres to the Strengthening the Reporting of Observational Studies in Epidemiology (STROBE) reporting guideline for observational studies. The research and analysis plan was preregistered.^31^ Patients were recruited using referral sampling from geographically dispersed mental health services across the Flanders region of Belgium. Inclusion criteria were (1) age between 15 and 39 years, (2) Dutch language proficiency, (3) past-month NSSI urges and/or behavior, and (4) receipt of treatment as usual. Data collection involved a survey battery and a clinical interview, followed by a 28-day EMA protocol comprising 6 regular semirandom EMA surveys approximately every 2 waking hours between 10 am and 9:30 pm. Additional burst surveys, triggered by intense urges,^17^ were not included in this report and were solely used to screen for NSSI behavior between regular surveys. A total of 132 participants were enrolled (eFigure 7 in Supplement 1), with adherence to the standard protocol assessed in 3 ways: (1) attrition (ie, proportion of patients who withdrew from the study) and daily engagement rate (ie, ≥1 survey daily), (2) adherence (ie, proportion of surveys completed vs the number received), and (3) number of valid assessments (ie, surveys submitted within 45 minutes once initiated). Participants provided written informed consent and received financial compensation,^17^ and the study procedures were approved by the Ethics Committee Research UZ/KU Leuven.

### Baseline Measures

Sociodemographic characteristics obtained, including patients’ age, gender (female, male, or nonbinary), sexual orientation (heterosexual, gay/lesbian, bisexual, or asexual), professional status (employed, unemployed, sick leave, student, or other), and educational level (elementary school, high school, or college/university) were assessed. The type of care included inpatient, outpatient, or hybrid (a combination) treatment as usual. The Structured Clinical Interview for *DSM-5* was used to assess mental disorders.^32^ Interrater reliability was excellent, with agreement rates ranging from 92% to 100% and a mean κ value of 0.95 (range, 0.84-1.00) in a 20% subsample (n = 25). The McLean Screening Instrument was used to screen for BPD, which exhibits good-to-excellent sensitivity and specificity (>0.80) for BPD, with a cutoff sum score of 7.^33^ The Self-Injurious Thoughts and Behaviors Interview was used to assess NSSI history, which previously showed strong construct validity (κ range, 0.74-1.00) and excellent test-retest reliability (κ range = 0.93-1.00).^34,35,36^ Nonsuicidal self-injury disorder was evaluated using *DSM-5* criteria.^37,38^

### Ecological Momentary Assessment

Nonsuicidal self-injury cognitions and behavior were assessed during each EMA survey via the m-Path app.^39^ Patients were asked to retrospectively rate the extent to which they experienced NSSI thoughts (“Since the last beep, have you considered deliberately hurting yourself without wanting to die?”) on a 7-point scale (not at all [0] to a lot [6]). Momentary NSSI urges were assessed (“Right now, how strong is the urge present to hurt yourself without wanting to die?”) on a 7-point scale (absent [0] to very strong [6]). Momentary perceived self-efficacy was assessed by asking patients how confident they felt in their ability to resist NSSI (“Right now, how confident are you that you can resist engaging in NSSI?”) on a 7-point scale (not at all [0] to very [6]). During each EMA survey, patients were asked whether they had self-injured (“Since the last beep, have you deliberately hurt yourself without wanting to die [for example, cut, scratched, or hit yourself]?)” using a dichotomous (no [0] or yes [1]) item. Patients were also instructed to register self-injury acts using an event marker in the app (available for 91.2% of participants). Nonsuicidal self-injury behavior was considered present between consecutive assessments whenever it was retrospectively reported in a survey or had been registered since the last completed survey.

### Statistical Analysis

Analyses were conducted in R, version 4.4.1 (R Foundation for Statistical Computing) and Mplus, version 8.3 (Muthén & Muthén). Time-invariant statistics were used to investigate the general short-term course and individual differences, including within-person proportions, modes, means, SDs, and intraclass correlation coefficients. To investigate variation within patients, metrics of measurement-to-measurement variability were examined, including the within-person root mean square of successive differences^40^ and the proportion of times in which the intensity of NSSI cognitions differed from previous ratings by more than 1 within-person SD^41^ This latter analysis was based on regular surveys less than 2 hours apart to capture rapid changes. For NSSI behavior, the proportion of times self-injury was present was calculated.

To investigate temporal variation within individuals, cycles (once and twice per day),^42^ within-day patterns (up to quintic function), trends across 28 days (up to cubic function), and dummies for days of the week and the daily EMA surveys were calculated.^43^ The highest degree term for polynomial functions was interpreted if it was significant. Bayesian multilevel vector-autoregressive models within the residual dynamic structural equation modeling framework were used (eMethods, eFigures 1-6, eTable 1 in Supplement 1).^44,45,46,47^ These models included random intercepts and slopes, with residual variances being person-specific when possible. Recommendations were followed to include individuals with at least 25 surveys for the specification of random effects.^48^ To investigate within-day variation for NSSI behavior, a dataset was constructed based on the event registrations, indicating the presence/absence of self-injury across 1-hour intervals. Individual distributions were visualized using ggplot2.^49^ Each model consisted of at least 2500 recorded iterations with thinning set to 20 to address potential autocorrelation between subsequent parameter values during estimation. In bayesian analysis, an estimate is considered significant if the 95% credibility interval (CrI) excludes 0 and the 1-sided *P* value represents the proportion of the posterior distribution either below 0 for positive estimates or above 0 for negative estimates.

## Results

### Adherence and Cohort Selection

Of the 132 enrolled participants (mean [SD] age, 22.82 [5.37] years; 86.36% female, 6.82% male, 6.82% nonbinary), 8 dropped out (attrition = 6.06%), and 124 completed surveys on 26.74 of 28 days (daily engagement rate = 95.50%). One of the 124 participants did not complete 25 surveys and was excluded from the cohort, while 2 of the 8 dropouts completed more than 25 surveys and could be included. This resulted in a cohort of 125 patients (94.70% of the initial sample) who completed a total of 15 123 surveys. The patients included 109 females (87.2%), 8 males (6.4%), and 8 nonbinary individuals (6.4%); 66 were heterosexual (52.8%). Median age was 22.0 (range, 15-39) years. The median adherence rate was 78.6% (IQR, 59.5%-88.7%), with a mean (SD) of 121 (34.5) surveys per patient and a median completion time of 86 (IQR, 61-127) seconds. There were 25 surveys submitted in total after 45 minutes, resulting in 15 098 valid assessments.

### Cohort Characteristics and Short-Term Course of NSSI

Table 1 presents demographic and clinical characteristics of the cohort. Most patients reported having engaged in NSSI more than 100 times in their lifetime (57.6%) and met criteria for *DSM-5* NSSI disorder (72.0%). Table 2 presents descriptive and variability statistics. Nonsuicidal self-injury thoughts and urges were present (EMA score >0) during most assessments (mean individual proportions, 56.84% for thoughts; 57.11% for urges), but were generally low in intensity (mean individual means, 1.57 for thoughts; 1.52 for urges) with moderate self-efficacy to resist self-injury (mean individual means, 4.38). Prevalence rates of NSSI behavior were estimated at 84.0% (monthly), 49.90% (weekly), and 18.19% (daily).

**Table 1.** Sociodemographic and Clinical Description of 125 Patients at Intake

Characteristic	No. (%)
**Sociodemographic**	
Developmental age groups, median (range)	22.0 (15-39)
Adolescents (15-18 y)	28 (22.4)
Emerging adults (19-29 y)	82 (65.6)
Adults (30-39 y)	15 (12.0)
Gender	
Female	109 (87.2)
Male	8 (6.4)
Nonbinary^a^	8 (6.4)
Sexual orientation	
Heterosexual	66 (52.8)
Gay/lesbian	16 (12.8)
Bisexual	40 (32.0)
Asexual	3 (2.4)
Highest educational level	
Primary school	26 (20.8)
Secondary school	71 (56.8)
College/university^b^	28 (22.4)
Professional status	
Sick leave (social benefits)	50 (40.0)
Studying	48 (38.4)
Employed	9 (7.2)
Unemployed	6 (4.8)
Other	12 (9.6)
**Clinical**	
Current *DSM-5* mental disorders	
Nonsuicidal self-injury disorder (past 12 mo)	90 (72.0)
Major depressive disorder (last month)	86 (68.8)
Generalized anxiety disorder (past 6 mo)	83 (66.4)
Posttraumatic stress disorder (last month)	71 (56.8)
Panic disorder (last month)	51 (40.8)
Eating disorder (past 3 mo)	48 (38.4)
Alcohol use disorder (past 12 mo)	33 (26.4)
Other substance use disorder (past 12 mo)	50 (40.0)
Any *DSM-5* mental disorder	124 (99.2)
No. of *DSM-5* mental disorders, median (range)	4.0 (0-7)
Screen borderline personality disorder	94 (75.2)
Mode of care	
Inpatient	45 (36.0)
Outpatient	45 (36.0)
Hybrid (combination)	35 (28.0)
Lifetime NSSI behavior, times	
11-20	4 (3.2)
21-50	24 (19.2)
51-100	25 (20.0)
>100	72 (57.6)
Top 3 prevalent NSSI methods	
Cut or carved skin	117 (93.6)
Scratched skin	94 (75.2)
Smashed hand/foot against wall or objects	89 (71.2)
Lifetime No. of methods, median (range)	7.0 (2-12)
Age of onset NSSI behavior, median (range)	14.0 (6-30)
Childhood (≤11 y)	16 (12.8)
Adolescence (12-18 y)	97 (77.6)
Emerging adulthood (19-29 y)	11 (8.8)
Adulthood (30-39 y)^c^	1 (0.8)
Past-year NSSI thoughts, times	
<5	0
5-20	7 (5.6)
21-50	19 (15.2)
51-100	28 (22.4)
>100	71 (56.8)
Past-year NSSI behavior, times	
<5^d^	7 (5.6)
5-20	30 (24.0)
21-50	38 (30.4)
51-100	28 (22.4)
>100	22 (17.6)
Past-month NSSI thoughts, times	
1-4	14 (11.2)
5-20	50 (40.0)
21-50	49 (39.2)
51-100	4 (3.2)
>100	8 (6.4)
Past-month NSSI behavior	107 (85.6)
Past-month times NSSI behavior, median (range)	5 (0-150)
Required medical treatment for NSSI	
No, but it was necessary	15 (12.0)
Yes	89 (71.2)
Uncontrollable urges present (strongly agree-agree)	101 (80.8)

Abbreviations: *DSM-5*, *Diagnostic and Statistical Manual of Mental Disorders, Fifth Edition, Text Revision*; NSSI, nonsuicidal self-injury.

^a^ Nonbinary includes transgender male-to-female (n = 1), transgender female-to-male (n = 2), and other category (n = 5).

^b^ College/university comprises individuals with a bachelor, master, or PhD degree.

^c^ One patient reported onset at age 30 years.

^d^ Two patients reported no NSSI behavior in the past year.

**Table 2.** Individual Descriptive and Variability Statistics of the Short-Term Course of NSSI

NSSI category	Mean of individual measurements across participants, (SD) [range]				ICC (95% CrI)^a^
NSSI cognitions					
Retrospective thoughts	Percentage across EMA surveys: 56.84 (33.92) [0-100]	Mode: 1.08 (1.60) [0-6]	Mean^b^: 1.57 (1.18) [0-5.24]	SD^c^: 1.26 (0.44) [0-2.20]	0.44 (0.38-0.51)
Momentary urge	Percentage across EMA surveys: 57.11 (33.84) [0-100]	Mode: 1.02 (1.54) [0-6]	Mean^b^: 1.52 (1.13) [0-5.11]	SD^c^: 1.24 (0.44) [0-2.27]	0.43 (0.37-0.50)
Self-efficacy to resist NSSI	Percentage across EMA surveys: 65.33 (33.18) [0-100]^d^	Mode: 4.81 (1.55) [0-6]	Mean^b^: 4.38 (1.13) [0.05-6.00]	SD^c^: 1.16 (0.41) [0-2.51]	0.47 (0.40-0.53)
NSSI behavior					
High-intensity urge^e^	Frequency across month: 17.90 (23.55) [0-135]	Frequency across week: 4.54 (6.56) [0-38]	Frequency across day: 0.67 (1.21) [0-6]	Percentage across regular EMA surveys: 15.67 (18.88) [0-91.82]	NA
NSSI behavior^f^	Frequency across month: 9.35 (17.17) [0-103]	Frequency across week: 2.37 (5.17) [0-42]	Frequency across day: 0.35 (1.07) [0-16]	NA	NA
NSSI behavior reported during surveys^g^	Frequency across month: 8.58 (16.75) [0-103]	Frequency across week: 2.18 (4.89) [0-41]	Frequency across day: 0.32 (1.01) [0-16]	Percentage across regular EMA surveys: 6.55 (10.27) [0-55.56]	NA
NSSI behavior reported via event marker^h^	Frequency across month: 2.32 (3.90) [0-26]	Frequency across week: 0.59 (1.67) [0-26]	Frequency across day: 0.09 (0.42) [0-12]	NA	NA
Prevalence of NSSI behavior, %	84.0	49.90	18.19	NA	NA

Abbreviations: CrI, credibility interval; EMA, ecological momentary assessment; ICC, intraclass correlation coefficient; NA, not applicable; NSSI, nonsuicidal self-injury.

^a^ Intraclass correlation coefficient represents the proportion of total variance accounted for by between-person variance.

^b^ Indicating mean of individual means.

^c^ Indicating mean of individual SDs.

^d^ Hesitation in one’s ability to resist NSSI is defined as a score of less than 6 on 0- to 6-point scale in regular EMA surveys.

^e^ A high-intensity urge is defined as a score of greater than or equal to 4 on 0- to 6-point scale in regular EMA surveys.

^f^ Individual mean frequencies were calculated based on the number of retrospectively reported NSSI behaviors across all available surveys and the unique number of event registrations within specified time window.

^g^ Individual mean frequencies were calculated based on the number of retrospectively reported NSSI behaviors across all available surveys within specified time window.

^h^ Information was available from 114 participants.

### Short-Term Course Variation Between Individuals

There was considerable between-patient variation (range of intraclass correlation coefficients, 0.43-0.47; range of individual means for cognitions, 0-6; and range of individual frequency behavior, 0-103) (Table 2). Nonsuicidal self-injury behavior occurred a mean (SD) of 9.35 (17.17) times, with variation in frequency ranging from 0 to 103 times (median, 3). Weekly occurrences ranged from 0 to 42 times (mean [SD] individual means, 2.37 [5.17] times), and daily occurrences ranged from 0 to 16 times (mean [SD] individual means, 0.35 [1.07] times). Adolescents reported more intense NSSI thoughts and urges than adults (slopes, 0.75-0.79; *P* ≤ .02), while patients who identified as gay or lesbian exhibited lower self-efficacy to resist self-injury than those who identified as heterosexual (slope, −0.70; 95% Crl, −1.31 to −0.07) (eTable 2 in Supplement 1). Patients diagnosed with posttraumatic stress disorder reported a higher intensity of thoughts and urges (adjusted slopes, 0.45-0.52; *P* ≤ .01), whereas those with NSSI disorder were more likely to self-injure (slope, 0.44; 95% Crl, 0.09-0.78) (eTable 3 in Supplement 1). The past-month frequency of thoughts at intake was associated with higher intensity of thoughts and urges (adjusted slopes, 0.72-1.13; *P* ≤ .002), lower self-efficacy (adjusted slopes, −0.62 to −0.78; *P* ≤ .006), and more frequent NSSI behavior during treatment (slopes, 0.65-0.92; *P* < .001). Patients with 10 or more past-month NSSI behaviors at intake showed the highest frequency of NSSI (slope, 1.56; 95% Crl, 1.08-2.12) (eTable 4 in Supplement 1).

### Short-Term Course Variation Within Individuals

**Figure 1.**
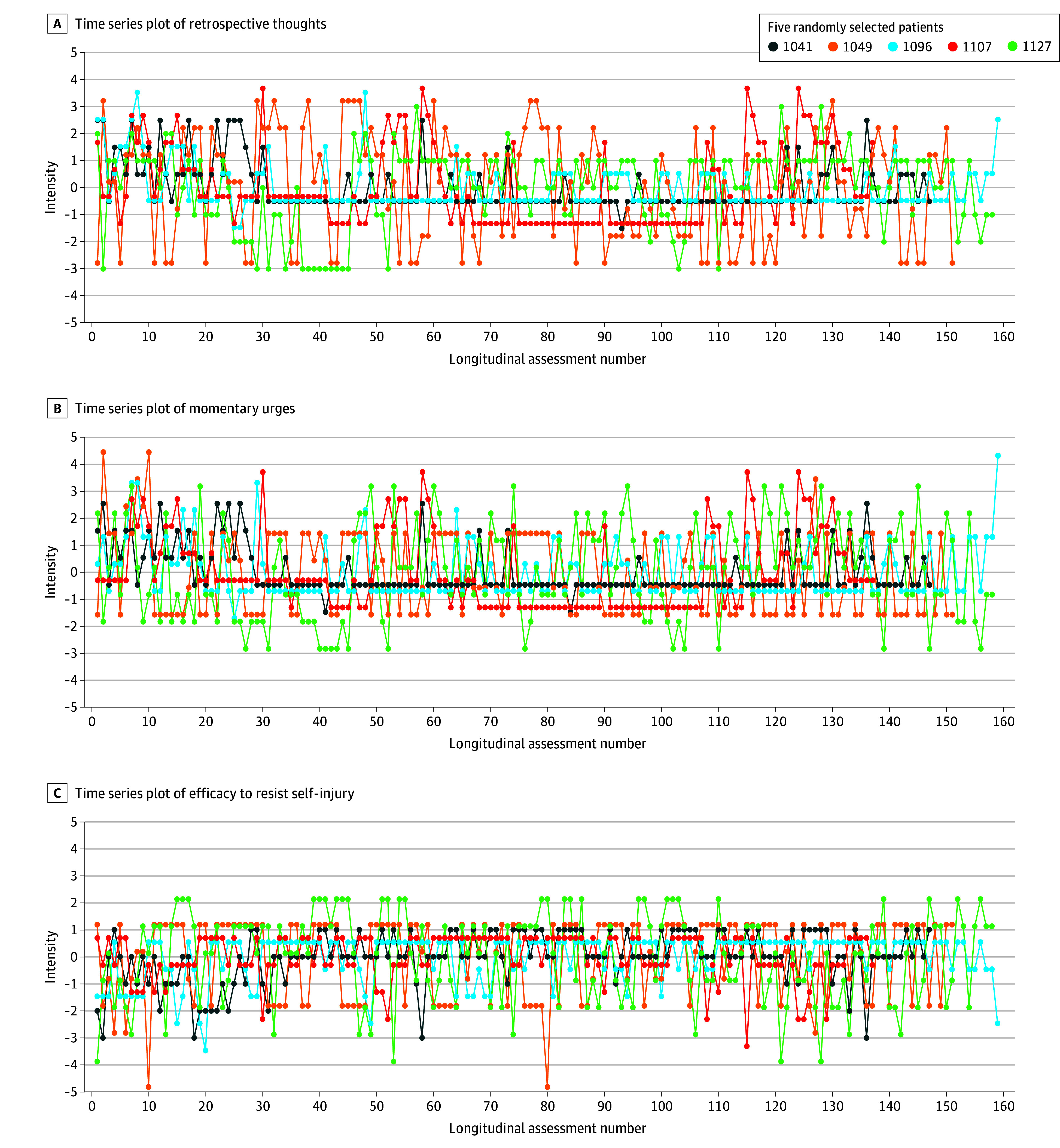
Within-Person Variability Across Randomly Selected Individuals Seeking Treatment for Nonsuicidal Self-Injury (NSSI) Times series plot of nonsuicidal self-injurious cognitions during 28 days of ecological momentary assessment (EMA). Retrospective NSSI thoughts (A), momentary NSSI urges (B), and efficacy to resist NSSI (C). Values are person-mean centered (comparing each patient’s hourly level of cognitions to their person-specific mean). The colored lines represent 5 randomly selected patients with adherence of 80% or more (ie, at least 134 assessments) to illustrate within-person variability on an hourly basis (median of 2.2 [IQR, 1.72-3.13] hours apart). eFigure 8 in Supplement 1 provides individual time series plots for each patient.

The root mean square of successive differences values revealed substantial within-patient instability in thoughts (mean [SD] individual means, 1.40 [0.51]), urges (mean [SD] individual means, 1.39 [0.49]), and self-efficacy (mean [SD] individual means, 1.31 [0.48]), indicating a sawtooth pattern over time (Figure 1). When considering 5096 surveys less than 2 hours apart (median interval, 93 [IQR, 74-107] minutes), ratings differed on average across patients by at least 1 SD in 23.33% of instances for thoughts, 22.43% for urges, and 23.85% for self-efficacy to resist NSSI. These changes were observed at least once for any NSSI cognition among 96% (range, 92.8%-95.2%) of patients. The median proportion of times NSSI behavior was present across all 15 098 assessments (median interval, 143 [IQR, 106-278] minutes) was 0.027 (IQR, 0.008-0.086). While no significant change in the intensity of thoughts and urges was observed during the entire EMA period (eTable 5 in Supplement 1), there was a linear increase in self-efficacy to resist self-injury (slope, 0.009; 95% CrI, 0.002-0.018) and a decrease in the propensity to self-injure (slope, −0.102; 95% CrI, −0.140 to −0.065). These trends were included in subsequent models. Retrospective NSSI thoughts demonstrated the highest intensity during the morning assessment (intercept, 1.596; 95% CrI, 1.390-1.808), exhibiting a significant decrease during the noon (slope = −0.032; 95% CrI, −0.054 to −0.011), early afternoon (slope, −0.024; 95% CrI, −0.045 to −0.002), and late afternoon (slope, −0.025; 95% CrI, −0.046 to −0.004) assessments. Nonsuicidal self-injury behavior was most likely to be reported retrospectively in the morning assessment (eTable 6 Supplement 1).

**Figure 2.**
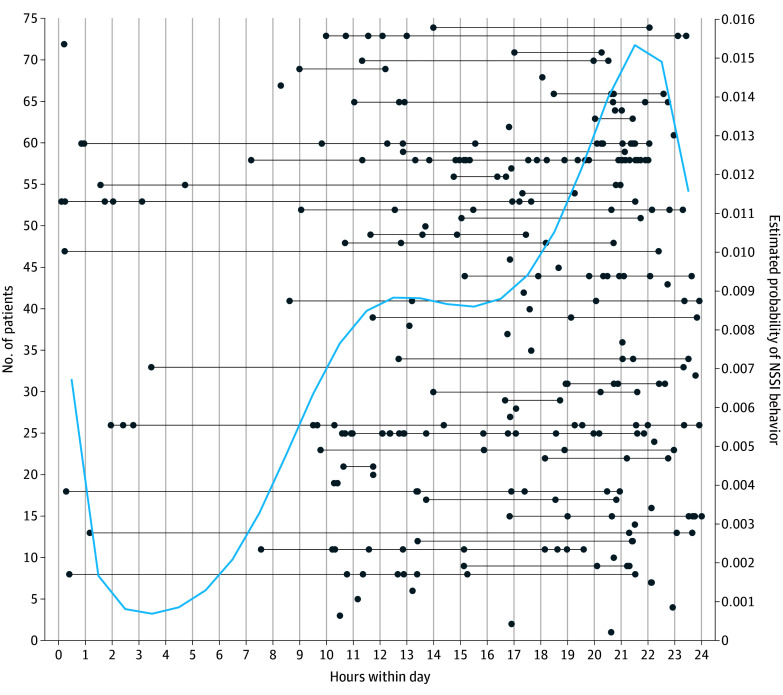
Within-Day Trajectory of Nonsuicidal Self-Injury (NSSI) Behavior Recorded with Event Marker Data indicate timing with event behavior; connected dots are events from the same patient. Individual distributions of NSSI behavior (black lines) and estimated probability across individuals (blue line; model 2 in the eMethods in Supplement 1 and quintic within-day trajectory in eTable 7 in Supplement 1). Probability is based on estimates after 2 weeks of monitoring (ie, in the middle of the study when the linear trend equals 0).

The timing of NSSI behaviors registered using the user-initiated event marker revealed a significant quintic risk trajectory (eTable 7 in Supplement 1), with a bimodal peak throughout the day (Figure 2). The probability of NSSI behavior was minimal between 1 and 5 am and increased from the morning hours to a first smaller peak around noon, after which the risk stabilized in the early afternoon until 4 pm. There was a steady increase from 6 pm until reaching a second, larger peak between 9 and 11 pm, followed again by a decrease into the night. Investigated cycles did not capture this trajectory (eFigure 9 in Supplement 1). Analyzing variation between days revealed a pattern in which the intensity of thoughts was lower (slope, −0.026; 95% CrI, −0.044 to −0.009), self-efficacy higher (adjusted slope, 0.045; 95% CrI, 0.023-0.070), and self-injury less likely (adjusted slope, −0.136; 95% CrI, −0.244 to −0.027) on Saturdays than other days (eTable 8 in Supplement 1).

### Associations Between NSSI Cognitions and Behavior

Nonsuicidal self-injury cognitions showed moderate to strong within-person contemporaneous correlations (range, −0.63 to 0.77) (Table 3). Nonsuicidal self-injury thoughts and urges exhibited positive temporal associations (range cross-regressive slopes, 0.31 to 0.49) and displayed negative bidirectional temporal associations with self-efficacy to resist self-injury (range cross-regressive slopes,  −0.10 to −0.36) over a 2-hour time scale. Controlling for the autoregressive association of NSSI behavior, higher-than-usual NSSI thoughts (cross-regressive slope = 0.31) and urges (cross-regressive slope = 0.31) and decreased self-efficacy to resist NSSI (cross-regressive slope = −0.30) were each associated with an increased risk of NSSI behavior. When considering all cognitions as lagged variables in a full multivariable regression, NSSI thoughts and self-efficacy uniquely estimated NSSI behavior within the next 2 hours (Table 3). Post hoc analyses revealed that these temporal associations did not differ between age groups (eTable 9 in Supplement 1).

**Table 3.** Contemporaneous and Temporal Associations Between NSSI Cognitions and Behavior

Cognition/behavior	Median point estimate (95% CrI)											
	Retrospective thoughts			Momentary urges			Self-efficacy to resist NSSI			NSSI behavior		
	Within-person contemporaneous and between-person correlations^a^^,^^b^	Outcome (lagged associations)^c^^,^^d^		Within-person contemporaneous and between-person correlations^a^^,^^b^	Outcome (lagged associations)^c^^,^^e^		Within-person contemporaneous and between-person correlations^a^^,^^b^	Outcome (lagged associations)^c^^,^^f^		Within-person contemporaneous and between-person correlations^a^^,^^b^	Outcome (lagged associations)^c^^,^^g^	
		Multivariable regression controlling for autoregressive association^h^	Full multivariable regression^i^		Multivariable regression controlling for autoregressive association^h^	Full multivariable regression^i^		Multivariable regression controlling for autoregressive association^h^	Full multivariable regression^i^		Multivariable regression controlling for autoregressive association^h^	Full multivariable regression^i^
Retrospective thoughts	NA	0.42 (0.38 to 0.47)^j^	0.02 (−0.02 to 0.06)	0.77 (0.77 to 0.78)^j^	0.37 (0.32 to 0.43)^j^	0.31 (0.26 to 0.37)^j^	−0.53 (−0.55 to −0.52)^j^	−0.25 (−0.26 to −0.18)^j^^,^^k^	−0.10 (−0.14 to −0.06)^j^	0.32 (0.24 to 0.39)^j^	0.31 (0.27 to 0.36)^j^	0.20 (0.13 to 0.28)^j^
Momentary urges	0.97 (0.95 to 0.98)^j^	0.49 (0.41 to 0.56)^j^	0.40 (0.32 to 0.48)^j^	NA	0.42 (0.38 to 0.46)^j^^,^^l^	0.02 (−0.02 to 0.05)	−0.63 (−0.64 to −0.62)^j^	−0.29 (−0.31 to −0.28)^b^^,^^k^	−0.17 (−0.21 to −0.13)^j^	0.24 (0.17 to 0.30)^j^	0.31 (0.27 to 0.36)^j^	0.06 (−0.01 to 0.15)
Self-efficacy to resist NSSI	−0.71 (−0.79 to −0.60)^j^	−0.36 (−0.43 to −0.28)^j^	−0.14 (−0.18 to −0.11)^j^	−0.75 (−0.82 to −0.65)^j^	−0.32 (−0.36 to −0.28)^j^	−0.22 (−0.26 to −0.18)^j^	NA	0.41 (0.37 to 0.45)^j^	0.16 (0.11 to 0.20)^j^	−0.15 (−0.24 to −0.02)^j^	−0.30 (−0.35 to −0.26)^j^	−0.12 (−0.19 to −0.06)^j^
NSSI behavior since last assessment	0.37 (0.19 to 0.53)^j^	0.01 (−0.10 to 0.13)	−0.03 (−0.12 to 0.06)	0.33 (0.13 to 0.48)^j^	0.02 (−0.07 to 0.11)	−0.13 (−0.20 to −0.06)^j^	−0.27 (−0.45 to −0.08)^j^	−0.07 (−0.08 to −0.05)^b^^,^^k^	0.10 (0.03 to 0.18)^j^	NA	0.33 (0.21 to 0.44)^j^	0.11 (0.01 to 0.20)^j^

Abbreviations: 95% CrI, 95% credibility interval; NA, not applicable; NSSI, nonsuicidal self-injury.

^a^ Within-person correlations between latent factors presented above the diagonal and between-person correlations below the diagonal, estimated within bivariate multilevel vector autoregressive models in a residual dynamic structural equation framework. Linear trends were included for self-efficacy to resist NSSI and NSSI behavior.

^b^ The n = 125; 15 098 assessments.

^c^ Within-person unstandardized lagged associations based on regular surveys with 2-hour intervals estimated within multilevel vector autoregressive models in a dynamic structural equation framework. Linear trends were included for self-efficacy to resist NSSI and NSSI behavior.

^d^ Patients with within-person variation in retrospective thoughts were included in the respective model; patients with variability in outcome, n = 124; assessments = 19 984.

^e^ Patients with within-person variation in momentary urges were included in the respective model; patients with variability in outcome, n = 124; assessments = 14 947.

^f^ Patients with within-person variation in self-efficacy to resist NSSI were included in the respective model; patients with variability in outcome, n = 123; assessments = 14 853.

^g^ Patients with within-person variation in NSSI behavior were included in the respective model; patients with variability in outcome, n = 105; assessments = 12 771.

^h^ These models include the lagged variables mentioned in the rows as a within-person cross-regressive parameter, together with the autoregressive parameter for the outcome under investigation.

^i^ These models include all the lagged variables mentioned in the rows jointly as within-person autoregressive and cross-regressive parameters for the outcome under investigation.

^j^ Indicates a 95% probability that the true value of the association is not null (ie, the CrI does not include 0).

^k^ This parameter had to be specified as a fixed slope (ie, without random effect) for the model to converge.

^l^ Covariances of random effects with residual variances had to be excluded for the model to converge.

## Discussion

There are 4 key findings from this study. The short-term course of NSSI (1) varies greatly between treatment-seeking individuals with past-month NSSI, (2) displays even greater variability within individuals, (3) shows greater risk toward the evening, and (4) can be estimated using patient characteristics and EMA. Each of these findings warrants further comment, and each holds implications for optimizing assessment strategies and the deployment of interventions.

Individuals with past-month NSSI seeking mental health care generally expressed low intensity of NSSI thoughts and urges and some doubt in their ability to resist self-injury across 28 days of self-monitoring with EMA, with daily rates of NSSI behavior close to 18%. However, considerable between-patient variability indicated that this course is heterogeneous, as 43% to 47% of changes in NSSI cognitions could be attributed to individual differences. While patients self-injured a mean of 9.35 times, there were also substantial individual differences (SD, 17.17). The recency and frequency of NSSI thoughts and behavior at intake were the most consistent baseline characteristics indicative of a more severe subsequent course during treatment. These findings underscore the relevance of a comprehensive NSSI assessment for professionals in the mental health field.^50^ Further research is required to confirm whether the identified sociodemographic (eg, more intense thoughts and urges among adolescents than adults) and clinical (eg, more frequent NSSI behavior among patients with NSSI disorder) characteristics are associated with the short-term course of NSSI during treatment.

A critical discovery is the highly dynamic nature of the short-term course of NSSI in this population. A major advantage of EMA is its ability to track intraindividual variation over time. This study observed that the greatest variation occurred within patients. There was low stability for NSSI cognitions, and NSSI behavior was irregularly present. Considerable within-person changes in intensity ratings for thoughts, urges, and self-efficacy were observed in more than 1 in 5 of instances between assessments less than 2 hours apart. These findings are consistent with another EMA study,^41^ which reported that more than one-quarter of intensity ratings for suicidal ideation differed by at least 1 SD from assessments 4 to 8 hours earlier. This underscores the value of EMA in capturing fluctuations in self-injurious thoughts among individuals seeking treatment.

To facilitate the deployment of interventions, we evaluated when NSSI behavior typically occurs and whether NSSI cognitions might have utility for signaling risk for NSSI behavior in the next 2 hours. Extending retrospective findings,^15,51^ event registrations of NSSI behavior were most likely to occur during the evening hours, with the highest risk observed between 9 and 11 pm. These results are consistent with observed within-day peaks for binge eating and vomiting,^52,53^ which are highly comorbid disordered-eating behaviors with similar functional contingencies.^54^ Nonsuicidal self-injury thoughts and diminished self-efficacy uniquely signaled risk for self-injury. Clinically, these results advocate for a person-centered approach to NSSI recovery, emphasizing the importance of associated cognitions, such as perceived self-efficacy in addressing ambivalence about resisting NSSI urges during treatment.^16,55,56^ Future research should enhance understanding of the transition from urges to behavior.

The present findings highlight the potential utility of EMA in capturing changes in the short-term course of NSSI and suggest that there might be a need to complement traditional treatment for self-injury with real-time assessments and interventions outside the clinical setting.^20,57^ Preliminary findings suggest the acceptability and potential of EMA and ecological momentary interventions,^58,59^ but this area remains underexplored for self-injury and suicide prevention.^60^ For instance, just-in-time adaptive interventions are a promising ecological momentary intervention for dynamic behaviors that would adapt the provision of the intervention when and where it is most needed and likely to be effective.^61^ Research is needed to explore the potential of ecological momentary interventions and just-in-time adaptive interventions for NSSI in treatment.^62^

### Limitations

The results of this study must be interpreted considering 4 important limitations. First, findings may be biased by confounding from unobserved determinants of the nonrandom probability of being in treatment. Second, despite a comprehensive assessment of the short-term course of NSSI with EMA, specific cognitions were assessed with single items. Third, the sample composition limits the generalizability of the findings to males, nonbinary individuals, early adolescents (ages 10-14 years), and non-Belgian care contexts, highlighting the need for further research. Fourth, regarding perceived self-efficacy, the present study does not exclude the possibility that individuals accurately perceive their ability to resist NSSI rather than low perceived self-efficacy, directly causing reduced regulatory effort. Future research with randomized targeted interventions must elucidate which of these explanations holds true. Similarly, a control group is required to determine whether the observed increase in self-efficacy and decrease in NSSI behavior is due to an EMA observation effect, treatment-related changes, or a combination of these factors.^63^ Finally, future studies should explore whether distinct NSSI cognition phenotypes can be identified that predict self-injury during treatment.^64^

## Conclusions

This study presents a detailed characterization of NSSI highlighting its heterogeneous and dynamic course among treatment-seeking individuals. This underscores the potential value of self-monitoring with EMA and emphasizes the need for real-time interventions that extend beyond the clinical setting, especially during the evening hours.

## Notes

Supplement 1. **eMethods.** Detailed Methods **eFigure 1.** Graphical Description of Model 0 and Model 1 **eFigure 2.** Graphical Description of Model 2 **eFigure 3.** Graphical Description of Model 3 **eFigure 4.** Graphical Description of Model 4 **eFigure 5.** Graphical Description of Model 5 **eFigure 6.** Graphical Description of Model 6 **eTable 1.** Dynamic Structural Equation Models in the Manuscript **eTable 2.** Sociodemographic Characteristics as Predictors of the Mean Intensity of Non-Suicidal Self-Injury Cognitions and the Occurrence of Behavior **eTable 3.** Mental Disorders and Mode of Care as Predictors of the Mean Intensity of Non-Suicidal Self-Injury Cognitions and the Occurrence of Behavior **eTable 4.** Non-Suicidal Self-Injury (NSSI) Characteristics as Predictors of the Mean Intensity of NSSI Cognitions and the Occurrence of Behavior **eTable 5.** Trends in the Course of Non-Suicidal Self-Injury Across 28 Days **eTable 6.** Daily Variation in the Course of Non-Suicidal Self-Injury **eTable 7.** Within-Day Trajectories of NSSI Behavior Using Timing of Event Marker **eTable 8.** Weekly Variation in the Course of Non-Suicidal Self-Injury **eTable 9.** Age-Specific Moderation of the Temporal Associations Between Non-Suicidal Self-Injury Cognitions and Behavior **eFigure 7.** Flowchart of Analytical Sample **eFigure 8.** One-Hundred Twenty-Five Individual Time Series Plots With Smoothed Line Curves for Non-Suicidal Self-Injury (NSSI) Cognitions **eFigure 9.** Daily Cycles for Non-Suicidal Self-Injury Behavior Using Timing of Event Marker

Supplement 2.
Data Sharing Statement

## References

[1] Gandhi A, Luyckx K, Baetens I, . Age of onset of non-suicidal self-injury in Dutch-speaking adolescents and emerging adults: an event history analysis of pooled data. Compr Psychiatry. 2018;80:170-178. doi:10.1016/j.comppsych.2017.10.007 29121554

[2] Kiekens G, Hasking P, Bruffaerts R, ; WHO World Mental Health International College Student (WMH-ICS) collaborators. Non-suicidal self-injury among first-year college students and its association with mental disorders: results from the World Mental Health International College Student (WMH-ICS) initiative. Psychol Med. 2023;53(3):875-886. doi:10.1017/S0033291721002245 34140062 PMC8683565

[3] Ose SO, Tveit T, Mehlum L. Non-suicidal self-injury (NSSI) in adult psychiatric outpatients—a nationwide study. J Psychiatr Res. 2021;133:1-9. doi:10.1016/j.jpsychires.2020.11.031 33296801

[4] Christensen K, Chu C, Silverman AL, Peckham AD, Björgvinsson T, Beard C. Prevalence and correlates of past-month suicidal thoughts, suicide attempts, and non-suicidal self-injury among adults in a partial hospital program. J Psychiatr Res. 2021;144:397-404. doi:10.1016/j.jpsychires.2021.10.022 34741837

[5] Millon EM, Alqueza KL, Kamath RA, . Non-suicidal self-injurious thoughts and behaviors among adolescent inpatients. Child Psychiatry Hum Dev. 2024;55(1):48-59. doi:10.1007/s10578-022-01380-1 35727385 PMC9782727

[6] Bresin K, Schoenleber M. Gender differences in the prevalence of nonsuicidal self-injury: a meta-analysis. Clin Psychol Rev. 2015;38:55-64. doi:10.1016/j.cpr.2015.02.009 25795294

[7] van Alphen NR, Stewart JG, Esposito EC, Pridgen B, Gold J, Auerbach RP. Predictors of rehospitalization for depressed adolescents admitted to acute psychiatric treatment. J Clin Psychiatry. 2017;78(5):592-598. doi:10.4088/JCP.15m10326 27529444 PMC5313382

[8] Ribeiro JD, Franklin JC, Fox KR, . Self-injurious thoughts and behaviors as risk factors for future suicide ideation, attempts, and death: a meta-analysis of longitudinal studies. Psychol Med. 2016;46(2):225-236. doi:10.1017/S0033291715001804 26370729 PMC4774896

[9] Kiekens G, Hasking P, Boyes M, . The associations between non-suicidal self-injury and first onset suicidal thoughts and behaviors. J Affect Disord. 2018;239:171-179. doi:10.1016/j.jad.2018.06.033 30014957

[10] Horwitz AG, Czyz EK, King CA. Predicting future suicide attempts among adolescent and emerging adult psychiatric emergency patients. J Clin Child Adolesc Psychol. 2015;44(5):751-761. doi:10.1080/15374416.2014.910789 24871489 PMC4247360

[11] American Psychiatric Association. Diagnostic and Statistical Manual of Mental Disorders. 5th ed, text revision. American Psychiatric Association; 2022.

[12] Muehlenkamp JJ, Tillotson V. Overview and epidemiology of NSSI in clinical samples. In: Lloyd-Richardson EE, Baetens I, Whitlock JL, eds. The Oxford Handbook of Nonsuicidal Self-Injury. Oxford University Press; 2024:127-148. doi:10.1093/oxfordhb/9780197611272.013.8

[13] Reichl C, Rockstroh F, Lerch S, Fischer-Waldschmidt G, Koenig J, Kaess M. Frequency and predictors of individual treatment outcomes (response, remission, exacerbation, and relapse) in clinical adolescents with nonsuicidal self-injury. Psychol Med. 2023;53(16):7636-7645. doi:10.1017/S0033291723001447 37282585 PMC10755228

[14] Fox KR, Huang X, Guzmán EM, . Interventions for suicide and self-injury: a meta-analysis of randomized controlled trials across nearly 50 years of research. Psychol Bull. 2020;146(12):1117-1145. doi:10.1037/bul0000305 33119344

[15] Turner BJ, Baglole JS, Chapman AL, Gratz KL. Experiencing and resisting nonsuicidal self-injury thoughts and urges in everyday life. Suicide Life Threat Behav. 2019;49(5):1332-1346. doi:10.1111/sltb.12510 30152181

[16] Hasking P, Whitlock J, Voon D, Rose A. A cognitive-emotional model of NSSI: using emotion regulation and cognitive processes to explain why people self-injure. Cogn Emot. 2017;31(8):1543-1556. doi:10.1080/02699931.2016.1241219 27702245

[17] Kiekens G, Claes L, Schoefs S, . The Detection of Acute Risk of Self-Injury Project: protocol for an ecological momentary assessment study among individuals seeking treatment. JMIR Res Protoc. 2023;12:e46244. doi:10.2196/46244 37318839 PMC10337382

[18] Shiffman S, Stone AA, Hufford MR. Ecological momentary assessment. Annu Rev Clin Psychol. 2008;4:1-32. doi:10.1146/annurev.clinpsy.3.022806.091415 18509902

[19] Myin-Germeys I, Kasanova Z, Vaessen T, . Experience sampling methodology in mental health research: new insights and technical developments. World Psychiatry. 2018;17(2):123-132. doi:10.1002/wps.20513 29856567 PMC5980621

[20] Kiekens G, Robinson K, Tatnell R, Kirtley OJ. Opening the black box of daily life in nonsuicidal self-injury research: with great opportunity comes great responsibility. JMIR Ment Health. 2021;8(11):e30915. doi:10.2196/30915 34807835 PMC8663644

[21] Gratch I, Choo TH, Galfalvy H, . Detecting suicidal thoughts: the power of ecological momentary assessment. Depress Anxiety. 2021;38(1):8-16. doi:10.1002/da.23043 32442349

[22] Esposito EC, Duan AM, Kearns JC, Kleiman EM, Conwell Y, Glenn CR. Measuring adolescents’ self-injurious thoughts and behaviors: comparing ecological momentary assessment to a traditional interview. Res Child Adolesc Psychopathol. 2022;50(8):1095-1105. doi:10.1007/s10802-022-00907-3 35254573

[23] Nock MK. Why do people hurt themselves? new insights into the nature and functions of self-injury. Curr Dir Psychol Sci. 2009;18(2):78-83. doi:10.1111/j.1467-8721.2009.01613.x 20161092 PMC2744421

[24] Fitzpatrick S, Kranzler A, Fehling K, Lindqvist J, Selby EA. Investigating the role of the intensity and duration of self-injury thoughts in self-injury with ecological momentary assessment. Psychiatry Res. 2020;284:112761. doi:10.1016/j.psychres.2020.112761 31951869

[25] Kiekens G, Hasking P, Nock MK, . Fluctuations in affective states and self-efficacy to resist non-suicidal self-injury as real-time predictors of non-suicidal self-injurious thoughts and behaviors. Front Psychiatry. 2020;11:214. doi:10.3389/fpsyt.2020.00214 32265760 PMC7099647

[26] Burke TA, Fox K, Kautz M, Siegel DM, Kleiman E, Alloy LB. Real-time monitoring of the associations between self-critical and self-punishment cognitions and nonsuicidal self-injury. Behav Res Ther. 2021;137:103775. doi:10.1016/j.brat.2020.103775 33421892

[27] Victor SE, Scott LN, Stepp SD, Goldstein TR. I want you to want me: interpersonal stress and affective experiences as within-person predictors of nonsuicidal self-injury and suicide urges in daily life. Suicide Life Threat Behav. 2019;49(4):1157-1177. doi:10.1111/sltb.12513 30159910 PMC6395579

[28] Hepp J, Carpenter RW, Freeman LK, Vebares TJ, Trull TJ. The environmental, interpersonal, and affective context of nonsuicidal self-injury urges in daily life. Personal Disord. 2021;12(1):29-38. doi:10.1037/per0000456 32881575 PMC7946409

[29] Zaki LF, Coifman KG, Rafaeli E, Berenson KR, Downey G. Emotion differentiation as a protective factor against nonsuicidal self-injury in borderline personality disorder. Behav Ther. 2013;44(3):529-540. doi:10.1016/j.beth.2013.04.008 23768678

[30] Houben M, Claes L, Vansteelandt K, Berens A, Sleuwaegen E, Kuppens P. The emotion regulation function of nonsuicidal self-injury: a momentary assessment study in inpatients with borderline personality disorder features. J Abnorm Psychol. 2017;126(1):89-95. doi:10.1037/abn0000229 27808541

[31] Kiekens G, Claes L, Kleiman E, . The short-term course of non-suicidal self-injury among individuals seeking psychiatric treatment. OSF. Published online June 6, 2023. doi:10.17605/OSF.IO/GT6QY PMC11581677 39436647

[32] First MB, Williams JBW, Karg RS, Spitzer RL, eds. User’s guide for the SCID-5-CV Structured Clinical Interview for DSM-5 disorders: Clinical Version. American Psychiatric Association; 2016.

[33] Zanarini MC, Vujanovic AA, Parachini EA, Boulanger JL, Frankenburg FR, Hennen J. A screening measure for BPD: the McLean Screening Instrument for Borderline Personality Disorder (MSI-BPD). J Pers Disord. 2003;17(6):568-573. doi:10.1521/pedi.17.6.568.25355 14744082

[34] Fox KR, Harris JA, Wang SB, Millner AJ, Deming CA, Nock MK. Self-Injurious Thoughts and Behaviors Interview–Revised: development, reliability, and validity. Psychol Assess. 2020;32(7):677-689. doi:10.1037/pas0000819 32324021

[35] Nock MK, Holmberg EB, Photos VI, Michel BD. Self-Injurious Thoughts and Behaviors Interview: development, reliability, and validity in an adolescent sample. Psychol Assess. 2007;19(3):309-317. doi:10.1037/1040-3590.19.3.309 17845122

[36] Gratch I, Fernandes SN, Bell KA, . Self-Injurious Thoughts and Behaviors Interview–Revised (SITBI-R): reliability, validity, and inter-informant agreement in an adolescent sample. J Clin Child Adolesc Psychol. 2022;51(4):484-494. doi:10.1080/15374416.2021.1901229 33847199 PMC8577009

[37] Kiekens G, Hasking P, Claes L, . The *DSM-5* nonsuicidal self-injury disorder among incoming college students: prevalence and associations with 12-month mental disorders and suicidal thoughts and behaviors. Depress Anxiety. 2018;35(7):629-637. doi:10.1002/da.22754 29697881

[38] Buelens T, Luyckx K, Bogaerts A, Raymaekers K, Claes L. Longitudinal development of non-suicidal self-injury disorder in adolescence: prospective prediction of stability and change by identity development, depression, trauma, and resilience. J Affect Disord. 2023;342:210-217. doi:10.1016/j.jad.2023.08.134 37690540

[39] Mestdagh M, Verdonck S, Piot M, . m-Path: an easy-to-use and highly tailorable platform for ecological momentary assessment and intervention in behavioral research and clinical practice. Front Digit Health. 2023;5:1182175. doi:10.3389/fdgth.2023.1182175 37920867 PMC10619650

[40] Revelle W. Psych: procedures for psychological, psychometric, and personality research. Accessed March 3, 2024. https://cran.r-project.org/web/packages/psych/index.html

[41] Kleiman EM, Turner BJ, Fedor S, Beale EE, Huffman JC, Nock MK. Examination of real-time fluctuations in suicidal ideation and its risk factors: results from two ecological momentary assessment studies. J Abnorm Psychol. 2017;126(6):726-738. doi:10.1037/abn0000273 28481571

[42] Flury BD, Levri EP. Periodic logistic regression. Ecology. 1999;80(7):2254-2260. doi:10.1890/0012-9658(1999)080[2254:PLR]2.0.CO;2

[43] Fisher AJ, Bosley HG. Identifying the presence and timing of discrete mood states prior to therapy. Behav Res Ther. 2020;128:103596. doi:10.1016/j.brat.2020.103596 32135317

[44] Asparouhov T, Hamaker EL, Muthén B. Dynamic structural equation models. Struct Equ Modeling. 2017;25(3):359-388. doi:10.1080/10705511.2017.1406803

[45] Hamaker EL, Asparouhov T, Muthén B. Dynamic structural equation modeling as a combination of time series modeling, multilevel modeling, and structural equation modeling. In: Hoyle RH, ed. The Handbook of Structural Equation Modeling. 2nd ed. Guildford Press; 2023.

[46] McNeish D, Somers JA, Savord A. Dynamic structural equation models with binary and ordinal outcomes in Mplus. Behav Res Methods. 2024;56(3):1506-1532. doi:10.3758/s13428-023-02107-3 37118647 PMC10611901

[47] McNeish D, Hamaker EL. A primer on two-level dynamic structural equation models for intensive longitudinal data in Mplus. Psychol Methods. 2020;25(5):610-635. doi:10.1037/met0000250 31855015

[48] Muthén B, Asparouhov T. Using Mplus to do dynamic structural equation modeling. Accessed March 3, 2023. https://www.statmodel.com/Webtalk6.shtml

[49] Wickham H. ggplot2: elegant graphics for data analysis. Springer-Verlag; 2016.

[50] Westers NJ, Tinsley B. Risk assessment, intervention, and guidance for first responders and medical settings. In: Lloyd-Richardson E, Baetens I, Whitlock J, eds. The Oxford Handbook of Nonsuicidal Self-Injury. Oxford University Press; 2024:873-893. doi:10.1093/oxfordhb/9780197611272.013.44

[51] Czyz EK, Glenn CR, Arango A, Koo HJ, King CA. Short-term associations between nonsuicidal and suicidal thoughts and behaviors: a daily diary study with high-risk adolescents. J Affect Disord. 2021;292:337-344. doi:10.1016/j.jad.2021.05.104 34139406 PMC8282747

[52] Lavender JM, Utzinger LM, Crosby RD, . A naturalistic examination of the temporal patterns of affect and eating disorder behaviors in anorexia nervosa. Int J Eat Disord. 2016;49(1):77-83. doi:10.1002/eat.22447 26282336 PMC5242485

[53] Forester G, Schaefer LM, Dodd DR, . Time-of-day and day-of-week patterns of binge eating and relevant psychological vulnerabilities in binge-eating disorder. Int J Eat Disord. 2023;56(9):1694-1702. doi:10.1002/eat.23995 37212510 PMC10600945

[54] Kiekens G, Claes L. Non-suicidal self-injury and eating disordered behaviors: an update on what we do and do not know. Curr Psychiatry Rep. 2020;22(12):68. doi:10.1007/s11920-020-01191-y 33037934 PMC7547297

[55] Lewis SP, Hasking PA. Self-injury recovery: a person-centered framework. J Clin Psychol. 2021;77(4):884-895. doi:10.1002/jclp.23094 33296508

[56] Andersson H, Svensson E, Magnusson A, Holmqvist R, Zetterqvist M. Young adults looking back at their experiences of treatment and care for nonsuicidal self-injury during adolescence: a qualitative study. Child Adolesc Psychiatry Ment Health. 2024;18(1):16. doi:10.1186/s13034-024-00706-2 38245758 PMC10800066

[57] Kleiman EM, Glenn CR, Liu RT. The use of advanced technology and statistical methods to predict and prevent suicide. Nat Rev Psychol. 2023;2(6):347-359. doi:10.1038/s44159-023-00175-y 37588775 PMC10426769

[58] Arshad U, Farhat-Ul-Ain, Gauntlett J, Husain N, Chaudhry N, Taylor PJ. A systematic review of the evidence supporting mobile- and internet-based psychological interventions for self-harm. Suicide Life Threat Behav. 2020;50(1):151-179. doi:10.1111/sltb.12583 31448847 PMC7027458

[59] Gromatsky M, Patel TA, Wilson SM, . Qualitative analysis of participant experiences during an ecological momentary assessment study of nonsuicidal self-injury among veterans. Psychiatry Res. 2022;310:114437. doi:10.1016/j.psychres.2022.114437 35183989 PMC9169428

[60] Coppersmith DDL, Dempsey W, Kleiman EM, Bentley KH, Murphy SA, Nock MK. Just-in-time adaptive interventions for suicide prevention: promise, challenges, and future directions. Psychiatry. 2022;85(4):317-333. doi:10.1080/00332747.2022.2092828 35848800 PMC9643598

[61] Nahum-Shani I, Smith SN, Spring BJ, . Just-in-time adaptive interventions (JITAIs) in mobile health: key components and design principles for ongoing health behavior support. Ann Behav Med. 2018;52(6):446-462. doi:10.1007/s12160-016-9830-8 27663578 PMC5364076

[62] Kleiman EM, Bentley KH, Glenn CR, Liu RT, Rizvi SL. Building on the past 50 years, not starting over: a balanced interpretation of meta-analyses, reviews, and commentaries on treatments for suicide and self-injury. Gen Hosp Psychiatry. 2022;74:18-21. doi:10.1016/j.genhosppsych.2021.11.002 34800775 PMC11290550

[63] Reininghaus U, Schwannauer M, Barne I, . Strategies, processes, outcomes, and costs of implementing experience sampling-based monitoring in routine mental health care in four European countries: study protocol for the IMMERSE effectiveness-implementation study. BMC Psychiatry. 2024;24(1):465. doi:10.1186/s12888-024-05839-4 38915006 PMC11194943

[64] Kleiman EM, Turner BJ, Fedor S, . Digital phenotyping of suicidal thoughts. Depress Anxiety. 2018;35(7):601-608. doi:10.1002/da.22730 29637663

