# Supplementary material for: The Short-Term Course of Nonsuicidal Self-Injury Among Individuals Seeking Psychiatric Treatment
Source: JAMA Netw Open. 2024 Oct 22;7(10):e2440510. doi: 10.1001/jamanetworkopen.2024.40510 (PMC11581677; doi:10.1001/jamanetworkopen.2024.40510)
Supplement: Supplement 1. — eMethods. Detailed Methods eFigure 1. Graphical Description of Model 0 and Model 1 eFigure 2. Graphical Description of Model 2 eFigure 3. Graphical Description of Model 3 eFigure 4. Graphical Description of Model 4 eFigure 5. Graphical Description of Model 5 eFigure 6. Graphical Description of Model 6 eTable 1. Dynamic Structural Equation Models in the Manuscript eTable 2. Sociodemographic Characteristics as Predictors of the Mean Intensity of Non-Suicidal Self-Injury Cognitions and the Occurrence of Behavior eTable 3. Mental Disorders and Mode of Care as Predictors of the Mean Intensity of Non-Suicidal Self-Injury Cognitions and the Occurrence of Behavior eTable 4. Non-Suicidal Self-Injury (NSSI) Characteristics as Predictors of the Mean Intensity of NSSI Cognitions and the Occurrence of Behavior eTable 5. Trends in the Course of Non-Suicidal Self-Injury Across 28 Days eTable 6. Daily Variation in the Course of Non-Suicidal Self-Injury eTable 7. Within-Day Trajectories of NSSI Behavior Using Timing of Event Marker eTable 8. Weekly Variation in the Course of Non-Suicidal Self-Injury eTable 9. Age-Specific Moderation of the Temporal Associations Between Non-Suicidal Self-Injury Cognitions and Behavior eFigure 7. Flowchart of Analytical Sample eFigure 8. One-Hundred Twenty-Five Individual Time Series Plots With Smoothed Line Curves for Non-Suicidal Self-Injury (NSSI) Cognitions eFigure 9. Daily Cycles for Non-Suicidal Self-Injury Behavior Using Timing of Event Marker [file jamanetwopen-e2440510-s001.pdf]

Supplementary Online Content

Kiekens G, Claes L, Kleiman E, et al. The Short-Term Course of Nonsuicidal self-injury among individuals seeking psychiatric treatment. *JAMA Netw Open*. 2024;7(10):e2440510. doi:10.1001/jamanetworkopen.2024.40510

eMethods. Detailed Methods

eFigure 1. Graphical Description of Model 0 and Model 1

eFigure 2. Graphical Description of Model 2

eFigure 3. Graphical Description of Model 3

eFigure 4. Graphical Description of Model 4

eFigure 5. Graphical Description of Model 5

eFigure 6. Graphical Description of Model 6

eTable 1. Dynamic Structural Equation Models in the Manuscript

eTable 2. Sociodemographic Characteristics as Predictors of the Mean Intensity of Non-Suicidal Self-Injury Cognitions and the Occurrence of Behavior

eTable 3. Mental Disorders and Mode of Care as Predictors of the Mean Intensity of Non-Suicidal Self-Injury Cognitions and the Occurrence of Behavior

eTable 4. Non-Suicidal Self-Injury (NSSI) Characteristics as Predictors of the Mean Intensity of NSSI Cognitions and the Occurrence of Behavior

eTable 5. Trends in the Course of Non-Suicidal Self-Injury Across 28 Days

eTable 6. Daily Variation in the Course of Non-Suicidal Self-Injury

eTable 7. Within-Day Trajectories of NSSI Behavior Using Timing of Event Marker

eTable 8. Weekly Variation in the Course of Non-Suicidal Self-Injury

eTable 9. Age-Specific Moderation of the Temporal Associations Between Non-Suicidal Self-Injury Cognitions and Behavior

eFigure 7. Flowchart of Analytical Sample

eFigure 8. One-Hundred Twenty-Five Individual Time Series Plots With Smoothed Line Curves for Non-Suicidal Self-Injury (NSSI) Cognitions

eFigure 9. Daily Cycles for Non-Suicidal Self-Injury Behavior Using Timing of Event Marker

This supplementary material has been provided by the authors to give readers additional information about their work.

© 2024 Kiekens G et al. *JAMA Network Open.*

eMethods. Detailed Methods

Dynamic Structural Equation Modeling

Dynamic Structural Equation Modeling (DSEM) integrates three distinct modeling approaches: time-series modeling, multilevel modeling, and structural equation modeling.[1-3] Time-series modeling is utilized to account for lagged autoregressive and cross-regressive associations in longitudinally intensive Ecological Momentary Assessment (EMA) data. Multilevel modeling addresses the hierarchical structure of the data (i.e., repeated assessments within individuals), enabling the examination of associations across participants while capturing variability both within and between individuals over time. Finally, structural equation modeling is incorporated to account for measurement error and to include multiple outcome, latent, and mediating variables. For a detailed description of DSEM, readers are referred to Hamaker and colleagues and McNeish and colleagues.[2-5]

Bayesian Estimation

Bayesian estimation was employed for all models using non-informative (diffuse) priors and the Markov Chain Monte Carlo (MCMC) Gibbs sampler with the Metropolis-Hasting algorithm.[1] A Bayesian approach allows for the specification of random effects, providing asymptotically equivalent results to those from maximum likelihood estimation if they were computationally feasible. Recommendations were followed to include individuals with at least 25 assessments, which is necessary for the specification of random slopes and residual variances.[6] NSSI cognitions, measured as continuous variables, were analyzed using Bayesian linear regression. In contrast, NSSI behaviors, measured as binary variables, were analyzed using Bayesian probit regression. A positive probit regression coefficient indicates an increased probability of NSSI behavior, with a larger magnitude signifying a more rapid increase in probability. Unstandardized point estimates were derived from the median of each parameter's posterior distribution to investigate lagged associations from one time point to the next on the original scale. Standardized point estimates from the median of each parameter's posterior distribution in Residual DSEM models were used to obtain contemporaneous correlations between NSSI thoughts, urges, self-efficacy to resist NSSI, and propensity to engage in NSSI behavior. RDSEM models, as regular DSEM models, include autoregressive associations but model this through a lagged association between the residuals.[7] This approach allows RDSEM to maintain its emphasis on contemporaneous relationships within a given time interval.

Convergence was assessed using the Gelman-Rubin Potential Scale Reduction (PSR) criterion.[8,9] A PSR value close to 1 suggests convergence, meaning that between-chain variation is small relative to within-chain variation. Two chains were used as default, with a minimum of 2,500 and a maximum of 50,0000 recorded iterations. Thinning was applied with a value of 20 to mitigate potential autocorrelation between successive parameter values, meaning every 20th iteration was retained for posterior inference. Statistical significance was determined by examining 95% credibility intervals for each point estimate, which provides a 95% probability that the true value is non-null. In Bayesian analysis, the p-value is interpreted as the proportion of the posterior distribution below zero for positive estimates and above zero for negative estimates. For a comprehensive discussion of Bayesian analysis in Mplus, see Muthén, Muthén, and Asparouhov.[10]

Unequal time intervals and missing data

EMA data typically results in unequally spaced data in time due to semi-random sampling, which leads to lagged results being averaged across varying time intervals if not accounted for (which has been called the lag problem).[11] To ensure time-equidistant intervals in the analyses,[1] the TINTERVALstatement in Mplus 8.3 was used with one- (i.e., for momentarily registered NSSI behavior with the event marker) and two-hour intervals (i.e., for NSSI cognitions and behavior during regular assessments). This procedure creates a new time variable measured in hours since the first assessment and inserts based on the defined metric missing data records when no observation is present for the EMA assessments (there were no missing observations for momentarily registered NSSI behavior with event sampling). DSEM uses a discrete time Kalman filter approach to handling missing data,[1,3] in which it predicts the next observation based on lagged predictors. In the absence of a missed assessment, the filter relies on its previous prediction to estimate the missing value. When an assessment is available, the filter updates its prediction to incorporate this information, thereby improving the estimation. This iterative updating process has been shown to maintain accuracy in case of missing data at random,[12,13] with simulation studies having shown that the quality of the estimation in DSEM is preserved when the amount of missing data is ≤ 85%.[1]

Model Specifications

In what follows, the first-order multilevel vector autoregressive (VAR[1]) models investigated in the manuscript are described in more detail. Graphical presentations relied on examples provided by Hamaker and colleagues,[3] and used NSSI thoughts (TH) as a prototypical example to illustrate how DSEM decomposes the intensive longitudinal EMA data into a within- and between-person part within a multilevel framework. Each of these

© 2024 Kiekens G et al. *JAMA Network Open.*

parts are modeled for each individual i at occasion t: a person-specific mean ($TH_i^{(b)}$) at the between-person model with a temporal deviation from that mean ($TH_{it}^{(w)}$) over time using a time-series model to account for lagged relationships at the within-person model. The individual means contain between-person variance and can be further modeled at the between-person level. An unrestricted covariance structure was specified for random effects of intercepts and slopes in univariate multilevel vector autoregressive models, with bivariate and multivariate multilevel vector autoregressive models allowing only intercepts of means to covary for convergence. DSEM models included random intercepts and slopes, with residual variances being person-specific. The eMethods figures below show the different models that were used with particular parameters of interest, which are highlighted in blue.

Model 0, shown in the left pane of eMethods Figure 1, estimated intra-class coefficients for NSSI thoughts, urges, and self-efficacy to resist NSSI, as detailed in Table 2 of the manuscript. Model 1, depicted in the right pane of eMethods Figure 1, examined between-person predictors related to sociodemographic characteristics (see eTable 1), mental disorders and mode of care (see eTable 2), and NSSI characteristics (see eTable 3) for NSSI thoughts, urges, self-efficacy to resist NSSI, and retrospectively reported NSSI behavior during consecutive assessments. Model 2, shown in eMethods Figure 2, assessed trends (see eTable 4), within-day variation (see eTables 5 and 6), and within-week variation (see eTable 7) in NSSI thoughts, urges, self-efficacy to resist NSSI, and NSSI behavior during consecutive assessments. Model 3, shown in eMethods Figure 3, explored correlations between random effects of NSSI cognitions and behavior during consecutive assessments using bivariate multilevel VAR models presented in Table 3 of the manuscript. Model 4, illustrated in eMethods Figure 4, evaluated temporal associations using bivariate multilevel VAR models, as detailed in Table 3 of the manuscript (i.e., multivariable regressions controlling for autoregressive associations). Model 5, depicted in eMethods Figure 5, examined temporal associations with multivariate multilevel VAR models, as also outlined in Table 3 of the manuscript (i.e., full multivariable regressions). Lastly, Model 6, illustrated in eMethods Figure 6, investigated age-specific moderation (i.e., adolescents vs. emerging adults and adults) of temporal associations using multivariate multilevel VAR models (this was evaluated per request of an anonymous reviewer). Table 1 of the eMethods provides the analytical expressions for these models. To ensure transparency, HTML output files containing all model specifications and results are available on the study's Open Science Framework page.[14]

eReferences

1. Asparouhov T, Hamaker EL, Muthén B. Dynamic structural equation models. *Struct Equ Modeling*. 2017;25(3):359-388. doi: 10.1080/10705511.2017.1406803
2. Hamaker EL, Asparouhov T, Brose A, Schmiedek F, Muthen B. At the frontiers of modeling intensive longitudinal data: dynamic structural equation models for the affective measurements from the COGITO study. *Multivariate Behav Res*. 2018;53(6):820-841. doi:10.1080/00273171.2018.1446819
3. Hamaker EL, Asparouhov T, Muthén B. Dynamic structural equation modeling as a combination of time series modeling, multilevel modeling, and structural equation modeling. In: Hoyle RH, ed. *The Handbook of Structural Equation Modeling (2nd edition)*. Guildford Press; 2023.
4.McNeish D, Hamaker EL. A primer on two-level dynamic structural equation models for intensive longitudinal data in Mplus. *Psychol Methods*. 2020;25(5):610-635. doi:10.1037/met0000250
5. McNeish D, Somers JA, Savord A. Dynamic structural equation models with binary and ordinal outcomes in Mplus. *Behav Res Methods*. 2024;56(3):1506-1532. doi:10.3758/s13428-023-02107-3
6. Muthén B, Asparouhov T. Using Mplus to do dynamic structural equation modeling. ttps://www.statmodel.com/Webtalk6.shtml
7. Asparouhov T, Muthén B. Comparison of models for the analysis of intensive longitudinal data. *Struct Equ Modeling*. 2020;27:275-297. doi:10.1080/10705511.2019.1626733
8. Gelman A., Rubin DB. Inference from Iterative Simulation Using Multiple Sequences. *Stat. Sci.* 1992;7(4):457-472. doi:10.1214/ss/1177011136
9. Gelman A., Carlin J. B., Stern H. S., Dunson DB, Vehtari A., Rubel DB. *Bayesian data analysis. (3rd edition).* Boca Raton: Chapman & Hall; 2014.
10. Muthén BO, Muthén LK, Asparouhov T. *Regression and mediation analysis using Mplus*. Muthén & Muthén; 2016.
11. Reichardt CS. Commentary: are three waves of data sufficient for assessing mediation? *Multivariate Behav Res*. 2011;46(5):842-51. doi:10.1080/00273171.2011.606740
12. Harvey PD. *Forecasting, structural time series models and the Kalman filter*. University Press; 1989.
13. Kalman RE. A new approach to linear filtering and prediction problems. *J. Basic Eng*: Transactions. 1960;82:35-45.
14. Kiekens G, Claes L, Kleiman E, et al. The short-term course of non-suicidal self-injury among individuals seeking psychiatric treatment. osf.io/gt6qy. Published August 27, 2024.

© 2024 Kiekens G et al. *JAMA Network Open.*

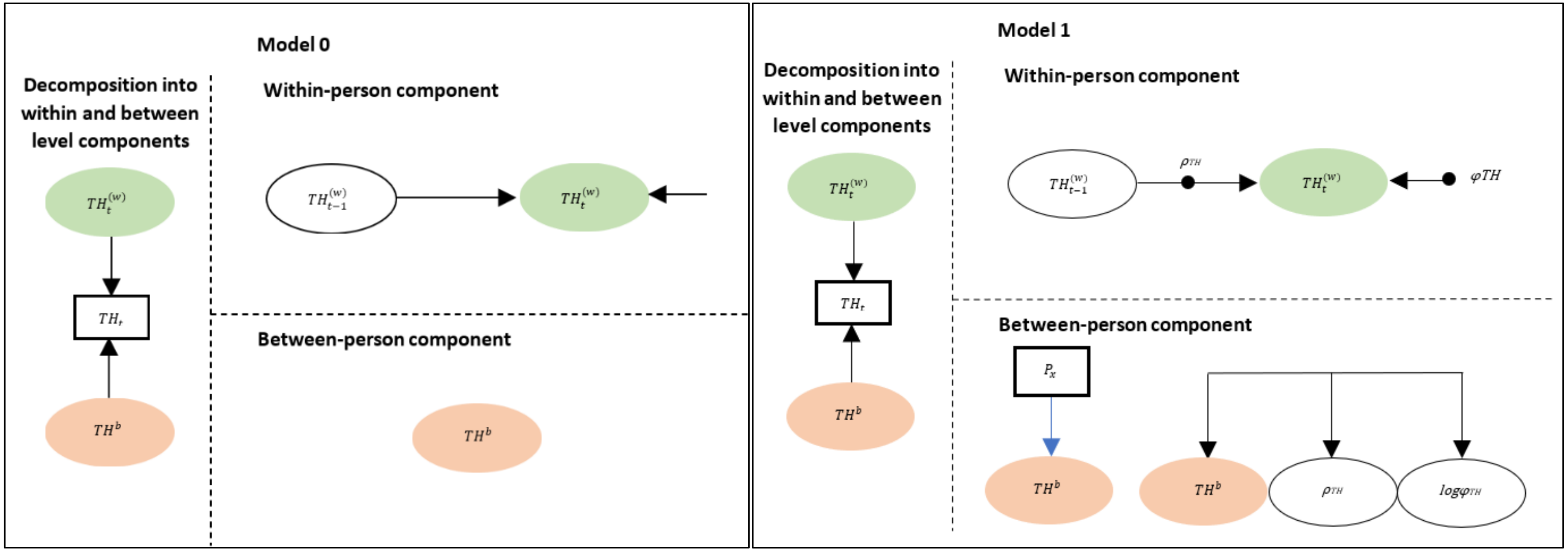

eFigure 1. Graphical Description of Model 0 (left) and Model 1 (right)

Note: Black dots represent random slopes and residual variances at the within-person level, corresponding to white circles at the between-person level. ρTH denotes autoregression over time for NSSI thoughts, and φTH indicates the residual variance. To prevent negative residual variances, the random effect of φTH is expressed as log(φTH) at the between-person level. Connected arrows at the between-person level indicate covariances between random effects. In the univariate multilevel VAR models with NSSI behavior, residual variances could not be estimated as random due to the categorical nature of the variable. Px represents the slopes of baseline characteristics on the between-person means (the parameter of interest in model 1, highlighted in blue). Significant sociodemographics were controlled for in models examining clinical characteristics as predictors of respective NSSI cognitions. Model 0 and model 1 included all participants.

© 2024 Kiekens G et al. *JAMA Network Open.*

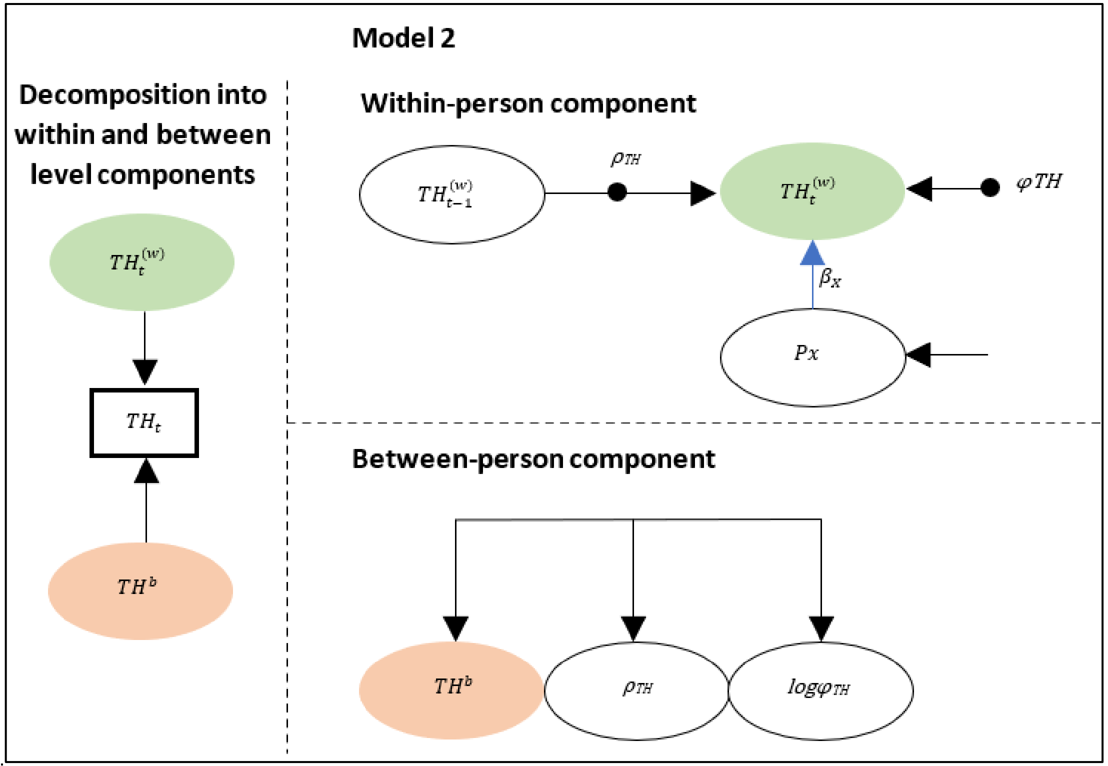

eFigure 2. Graphical Description of Model 2

Note: Black dots represent random slopes and residual variances at the within-person level, corresponding to white circles at the between-person level. ρTH denotes autoregression over time for NSSI thoughts, and φTH indicates the residual variance. The arrow from Px represents the slopes of observed time variables (parameter of interest; highlighted in blue), which had a non-random variance at the between-person level for the two-hourly consecutive assessments. The highest degree term for polynomial functions was interpreted if it had a 95% probability of being non-null. In the univariate multilevel VAR models with NSSI behavior, residual variances could not be estimated as random due to the categorical nature of the variable. This model included all participants with within-person variation in the outcome under investigation (here NSSI thoughts). One participant had data collection interrupted for 11 days after an initial 14 days due to a summer youth camp where smartphone use was prohibited. Only the first two weeks of this participant's data were included in the trend analyses across 28 days.

© 2024 Kiekens G et al. *JAMA Network Open.*

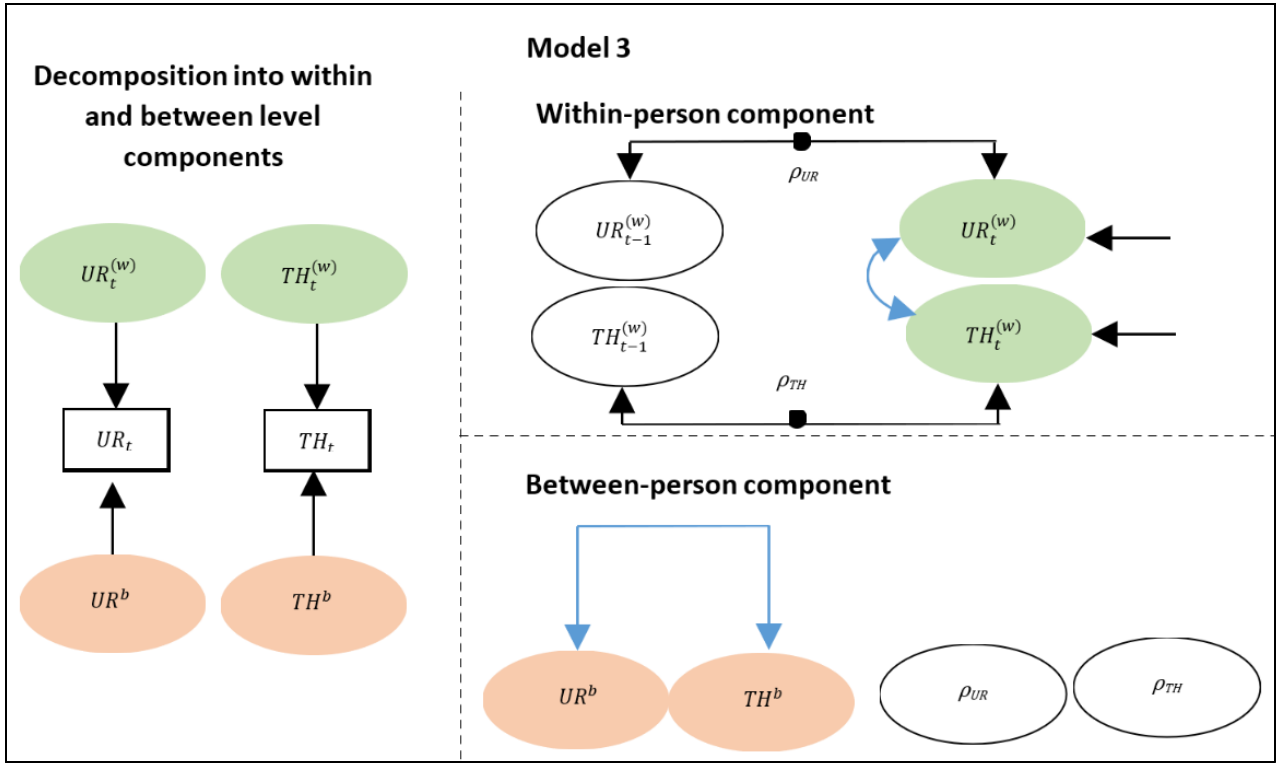

eFigure 3. Graphical Description of Model 3

Note: This is the only model that relies on Residual DSEM (RDSEM). The between-person part of the RDSEM model is the same as that of a regular DSEM model. The within-person model is separated into a 'structural part ' and 'autoregressive part', which models the autoregressive relationships via the residuals. This allows to preserve the focus of the model on contemporaneous relationships. Black dots represent random slopes at the within-person level, corresponding to white circles at the between-person level. ρUR/ρTH denotes the autoregression over time of NSSI urges/thoughts, respectively. Connected arrows indicate correlations with standardized coefficients. Linear trends were included for self-efficacy to resist NSSI and NSSI behavior in these models (cf. Model 2). This model included all participants.

© 2024 Kiekens G et al. *JAMA Network Open.*

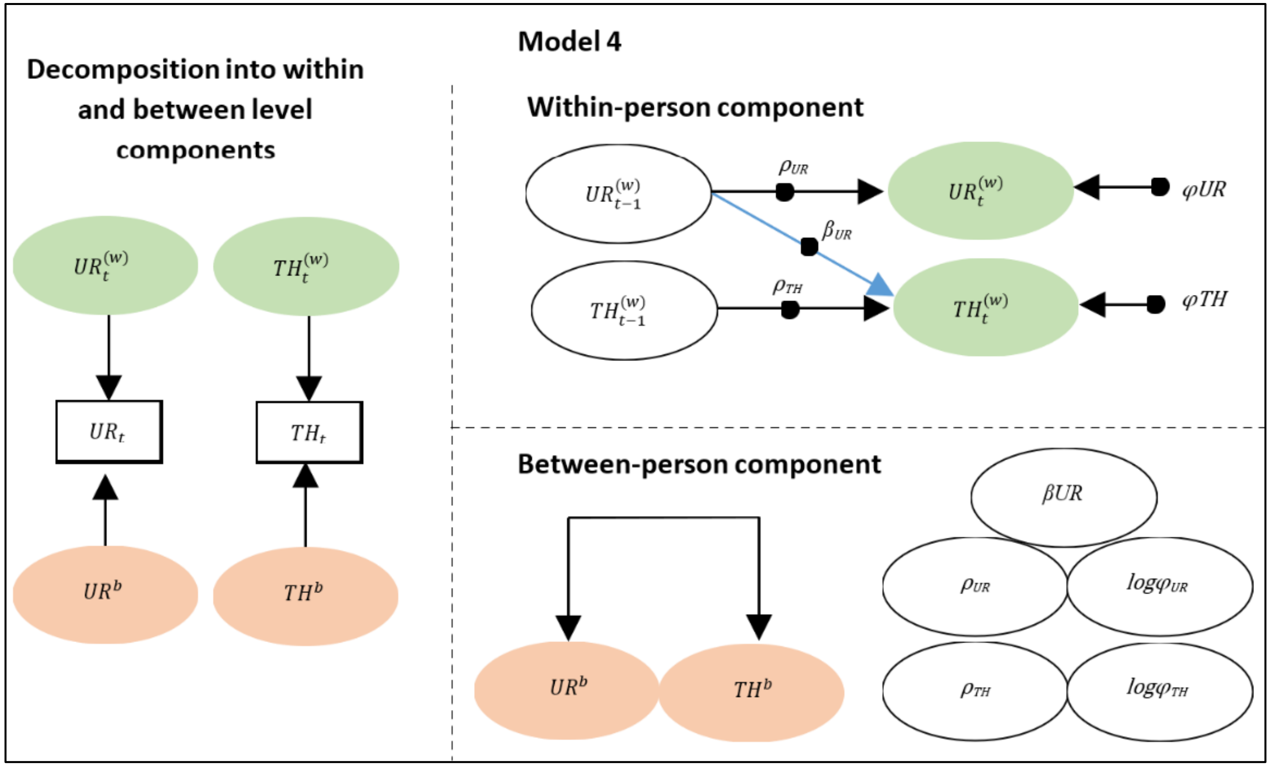

eFigure 4. Graphical Description of Model 4

Note: The figure depicts a prototypical example of momentary NSSI urges predicting retrospective NSSI thoughts at the next point. Black dots represent random slopes and residual variances at the within-person level, corresponding to white circles at the between-person level. ρUR/ρTH denotes the autoregression over time, while φUR/φTH indicates the residual variance. β_{UR} represents the lagged cross-regressive slope of momentary NSSI urges on NSSI thoughts at the next time point (parameter of interest; highlighted in blue), which has a random variance at the between-person level. To prevent negative residual variances, the random effect of φUR/φTH is expressed as log(φUR/φTH) at the between-person level. Connected arrows at the between-person level indicate covariances between random means. In bivariate multilevel VAR models with NSSI behavior, residual variances of behavior could not be estimated as random due to the categorical nature of the variable. Note that in Table 3 of the manuscript, autoregressive associations in univariate multilevel VAR models simplify to model 0 with the addition of random slopes and residual variances and covariances between random factors. Linear trends were included for self-efficacy to resist NSSI and NSSI behavior in these models (cf. Model 2). This model included all participants with within-person variation in the outcome under investigation (here NSSI thoughts).

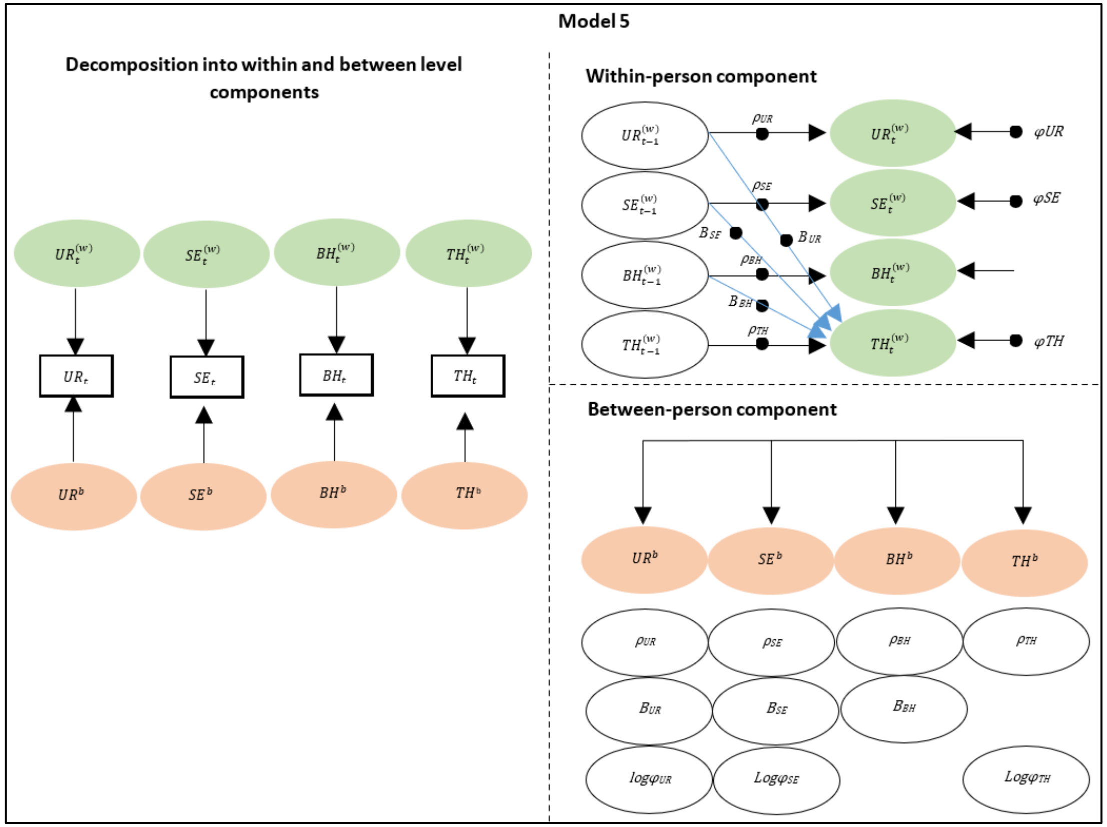

eFigure 5. Graphical Description of Model 5

Note: The figure depicts a prototypical example with momentary NSSI urges, self-efficacy to resist NSSI, and NSSI behavior predicting retrospective NSSI thoughts at the next time point (i.e., full multivariate regression with thoughts as outcome in Table 3 of the manuscript). Black dots represent random slopes and residual variances at the within-person level, corresponding to white circles at the between-person level. ρy denotes the autoregression over time, while φy represents the residual variance. β_y represents the lagged cross-regressive slopes on NSSI thoughts at the next time point (parameters of interest; highlighted in blue). To prevent negative residual variances, the random effect of φy is expressed as log(φy) at the between-person level. Connected arrows at the between-person level indicate covariances between random means. Linear trends were included for self-efficacy to resist NSSI and NSSI behavior in these models (cf. Model 2). This model included all participants with within-person variation in the outcome under investigation (here NSSI thoughts).

© 2024 Kiekens G et al. *JAMA Network Open.*

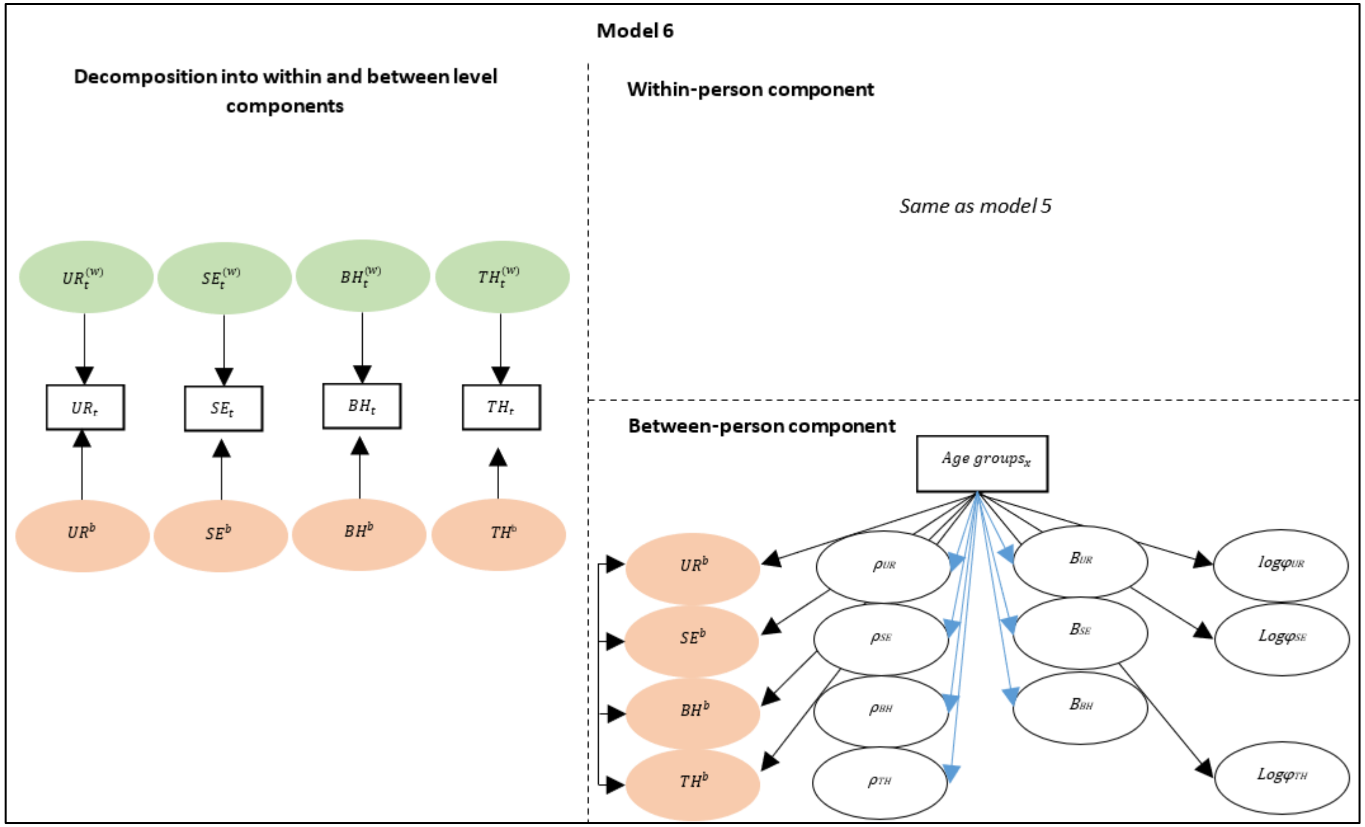

eFigure 6. Graphical Description of Model 6

Note: ρy denotes the autoregression over time, while φy indicates the residual variance. β_y represents the slopes of age groups with adolescents as the reference group on the random effects at the between-person level (estimates on autoregressive and crossregressive variance are parameters of interest; highlighted in blue). To prevent negative residual variances, the random effect of φy is expressed as log(φy) at the between-person level. Connected arrows at the between-person level indicate covariances between random means. Linear trends were included for self-efficacy to resist NSSI and NSSI behavior in these models (cf. Model 2). This model included all participants with within-person variation in the outcome under investigation (here NSSI thoughts).

eTable 1. Dynamic Structural Equation Models in the manuscript

Model	Within Expression	Between Expression
0	$TH_{it}^{(w)} = \rho_{TH} TH_{it-1}^{(w)} + \zeta_{THt}$	$TH_i^{(b)} = \gamma_{TH00} + u_{TH0i}$
1	$TH_{it}^{(w)} = \rho_{THi} TH_{it-1}^{(w)} + \zeta_{THit}$	$TH_i^{(b)} = \gamma_{TH00} + \gamma_{TH0x} p_{xi} + u_{TH0i}$<br>$\rho TH_i = \gamma_{TH1} + u_{TH1i}$<br>$\log(\varphi_{THi}) = \gamma_{TH2} + u_{TH2i}$
2	$TH_{it}^{(w)} = \rho_{THi} TH_{it-1}^{(w)} + \beta_X p_{xt}^{(w)} + \zeta_{THit}$	$TH_i^{(b)} = \gamma_{TH00} + + u_{TH0i}$<br>$\rho TH_i = \gamma_{TH1} + u_{TH1i}$<br>$\log(\varphi_{THi}) = \gamma_{TH2} + u_{TH2i}$
3	$UR_{it}^{(w)} = UR_{it}^{(w)} + \zeta_{URit}$<br>$\zeta_{URit} = \rho_{URi} \zeta_{it-1}^{(w)} + \delta_{it}$<br>$TH_{it}^{(w)} = TH_{it}^{(w)} + \zeta_{THit}$<br>$\zeta_{THit} = \rho_{THi} \zeta_{it-1}^{(w)} + \delta_{it}$	$UR_i^{(b)} = \gamma_{UR0} + u_{UR0i}$<br>$TH_i^{(b)} = \gamma_{TH0} + u_{TH0i}$<br>$\rho UR_i = \gamma_{UR1} + u_{UR1i}$<br>$\rho TH_i = \gamma_{TH1} + u_{TH1i}$
4	$UR_{it}^{(w)} = \rho_{URi} UR_{it-1}^{(w)} + \zeta_{URit}$<br>$TH_{it}^{(w)} = \rho_{THi} TH_{it-1}^{(w)} + \beta_{UR} UR_{it-1}^{(w)} + \zeta_{THit}$	$UR_i^{(b)} = \gamma_{UR0} + u_{UR0i}$<br>$TH_i^{(b)} = \gamma_{TH0} + u_{TH0i}$<br>$\rho UR_i = \gamma_{UR1} + u_{UR1i}$<br>$\rho TH_i = \gamma_{TH1} + u_{TH1i}$<br>$\beta UR_i = \gamma_{UR2} + u_{UR2i}$<br>$\log(\varphi UR_i) = \gamma_{UR3} + u_{UR3i}$<br>$\log(\varphi TH_i) = \gamma_{TH3} + u_{TH3i}$
5	$UR_{it}^{(w)} = \rho_{URi} UR_{it-1}^{(w)} + \zeta_{URit}$<br>$SE_{it}^{(w)} = \rho_{SEi} SE_{it-1}^{(w)} + \zeta_{SEit}$<br>$BH_{it}^{(w)} = \rho_{BHi} BH_{it-1}^{(w)} + \zeta_{BHit}$<br>$TH_{it}^{(w)} = \rho_{THi} TH_{it-1}^{(w)} + \beta_{UR} UR_{it-1}^{(w)}$<br>$+ \beta_{SE} SE_{it-1}^{(w)} + \beta_{BH} BH_{it-1}^{(w)} + \zeta_{THit}$	$UR_i^{(b)} = \gamma_{UR0} + u_{UR0i}$<br>$SE_i^{(b)} = \gamma_{SE0} + u_{SE0i}$<br>$BH_i^{(b)} = \gamma_{BH0} + u_{BH0i}$<br>$TH_i^{(b)} = \gamma_{TH0} + u_{TH0i}$<br>$\rho UR_i = \gamma_{UR1} + u_{UR1i}$<br>$\rho SE_i = \gamma_{SE1} + u_{SE1i}$<br>$\rho BH_i = \gamma_{BH1} + u_{BH1i}$<br>$\rho TH_i = \gamma_{TH1} + u_{TH1i}$<br>$\beta UR_i = \gamma_{UR2} + u_{UR2i}$<br>$\beta SE_i = \gamma_{SE2} + u_{SE2i}$<br>$\beta BH_i = \gamma_{BH2} + u_{BH2i}$<br>$\log(\varphi UR_i) = \gamma_{UR3} + u_{UR3i}$<br>$\log(\varphi SE_i) = \gamma_{SE3} + u_{SE3i}$<br>$\log(\varphi TH_i) = \gamma_{TH3} + u_{TH3i}$
6	Same as model 5	$UR_i^{(b)} = \gamma_{UR00} + \gamma_{UR01} Age_{emergingadults_i} +$<br>$\gamma_{UR02} Age_{adults_i} + u_{UR0i}$<br>$SE_i^{(b)} = \gamma_{SE00} + \gamma_{SE01} Age_{emergingadults_i} +$<br>$\gamma_{SE02} Age_{adults_i} + u_{SE0i}$<br>$BH_i^{(b)} = \gamma_{BH00} + \gamma_{BH01} Age_{emergingadults_i} +$<br>$\gamma_{BH02} Age_{adults_i} + u_{BH0i}$<br>$TH_i^{(b)} = \gamma_{TH00} + \gamma_{TH01} Age_{emergingadults_i} +$<br>$\gamma_{TH02} Age_{adults_i} + u_{TH0i}$<br>$\rho UR_i = \gamma_{UR10} + \gamma_{UR11} Age_{emergingadults_i} +$<br>$\gamma_{UR12} Age_{adults_i} + u_{UR1i}$<br>$\rho SE_i = \gamma_{SE10} + \gamma_{SE11} Age_{emergingadults_i} +$<br>$\gamma_{SE12} Age_{adults_i} + u_{SE1i}$<br>$\rho BH_i = \gamma_{BH10} + \gamma_{BH11} Age_{emergingadults_i} +$<br>$\gamma_{BH12} Age_{adults_i} + u_{BH1i}$<br>$\rho TH_i = \gamma_{TH10} + \gamma_{TH11} Age_{emergingadults_i} +$<br>$\gamma_{TH12} Age_{adults_i} + u_{TH1i}$

© 2024 Kiekens G et al. *JAMA Network Open.*

Model	Within Expression	Between Expression
6 (cont.)		$\beta UR_i = \gamma_{UR20} + \gamma_{UR21} Age_{emergingadults_i} + \gamma_{UR22} Age_{adults_i} + u_{UR2i}$<br>$\beta SE_i = \gamma_{SE20} + \gamma_{SE21} Age_{emergingadults_i} + \gamma_{SE22} Age_{adults_i} + u_{SE2i}$<br>$\beta BH_i = \gamma_{BH20} + \gamma_{BH21} Age_{emergingadults_i} + \gamma_{BH22} Age_{adults_i} + u_{BH2i}$<br>$\log(\varphi UR_i) = \gamma_{UR30} + \gamma_{UR31} Age_{emergingadults_i} + \gamma_{UR32} Age_{adults_i} + u_{UR3i}$<br>$\log(\varphi SE_i) = \gamma_{SE30} + + \gamma_{BH31} Age_{emergingadults_i} + \gamma_{BH32} Age_{adults_i} + u_{SE3i}$<br>$\log(\varphi TH_i) = \gamma_{TH30} + \gamma_{TH31} Age_{emergingadults_i} + \gamma_{TH32} Age_{adults_i} + u_{TH3i}$

Note: Similar models were run for NSSI urges, self-efficacy to resist NSSI, and NSSI behavior. For the models predicting NSSI behavior, the only difference is that the residual variances could not be estimated as random, as indicated in the graphical descriptions.

© 2024 Kiekens G et al. *JAMA Network Open.*

eTable 2. Sociodemographic Characteristics as Predictors of the Mean Intensity of Non-Suicidal Self-Injury Cognitions and the Occurrence of Behavior

	Proportion sample (n=125)	Mean Intensity Retrospective thoughts β (95% CrL)	Mean Intensity Momentary urges β (95% CrL)	Mean Intensity self-efficacy to resist NSSI β (95% CrL)	Mean Propensity NSSI behavior β (95% CrL)
Developmental age group models:					
Adolescents (15-18 years)	.224	Reference	Reference	Reference	Reference
Emerging adults (19-29 years)	.656	-0.34 (-0.81, 0.14)	-0.29 (-0.73, 0.17)	0.01 (-0.46, 0.52)	-0.32 (-0.68, 0.03)
Adults (30-39 years)	.120	**-0.75 (-1.46, -0.07)**	**-0.79 (-1.45, -0.13)**	0.37 (-0.33, 1.09)	0.09 (-0.39, 0.61)
Gender models:					
Female	.872	Reference	Reference	Reference	Reference
Male	.064	-0.58 (-1.42, 0.31)	-0.52 (-1.29, 0.29)	-0.47 (-1.28, 0.35)	-0.45 (-1.16, 0.23)
Non-binary[a]	.064	-0.05 (-0.86, 0.74)	-0.10 (-0.90, 0.67)	0.14 (-0.69, 0.93)	0.06 (-0.53, 0.65)
Sexual orientation models:					
Heterosexual	.528	Reference	Reference	Reference	Reference
Gay/lesbian	.128	0.59 (-0.01, 1.20)	0.58 (-0.02, 1.18)	**-0.70 (-1.31, -0.07)**	-0.12 (-0.59, 0.34)
Bisexual/asexual	.344	0.14 (-0.28, 0.56)	0.10 (-0.31, 0.51)	0.08 (-0.35, 0.51)	-0.09 (-0.39, 0.24)

Note: Each sociodemographic characteristic displays the result of a separate vector autoregressive model within a dynamic structural equation model (model 1 in eMethods) with the independent variable(s) specified in the row as a between-person predictor and the NSSI variable in the columns as outcome (i.e., random effect of means). Boldface for within-person associations indicates a 95% probability that the true value of the association is not null (i.e., the credibility interval does not include zero). [a] non-binary includes transgender male-to-female (n=1), transgender female-to-male (n=2), and the other category (n=5). β = Median Point Estimate, 95% CrI = 95% Credibility Interval, NSSI = Non-Suicidal Self-Injury.

© 2024 Kiekens G et al. *JAMA Network Open.*

eTable 3. Mental Disorders and Mode of Care as Predictors of the Mean Intensity of Non-Suicidal Self-Injury Cognitions and the Occurrence of Behavior

	Proportion sample (n=125)	Mean Intensity Retrospective thoughts β (95% CrL)	Mean Intensity Momentary urges β (95% CrL)	Mean Intensity self-efficacy to resist NSSI β (95% CrL)	Mean Propensity NSSI behavior β (95% CrL)
Current DSM-5 Mental Disorders:					
Non-suicidal self-injury disorder models	.720	0.14 (-0.32, 0.59)	0.09 (-0.32, 0.53)	-0.15 (-0.58, 0.30)	**0.44 (0.09, 0.78)**
Major depressive disorder models	.688	-0.26 (-0.69, 0.14)	-0.14 (-0.52, 0.27)	0.13 (-0.28, 0.56)	0.21 (-0.10, 0.54)
Generalized anxiety disorder models	.664	0.28 (-0.12, 0.67)	0.37 (-0.01, 0.76)	-0.08 (-0.49, 0.33)	-0.11 (-0.43, 0.21)
Post-traumatic stress disorder models	.568	**0.45 (0.06, 0.83)**	**0.52 (0.17, 0.87)**	-0.32 (-0.71, 0.08)	0.02 (-0.28, 0.33)
Panic disorder models	.408	-0.27 (-0.68, 0.13)	-0.12 (-0.49, 0.25)	0.37 (-0.01, 0.76)	-0.25 (-0.56, 0.06)
Eating disorder models	.384	-0.19 (-0.61, 0.22)	-0.09 (-0.46, 0.31)	0.12 (-0.26, 0.51)	-0.02 (-0.34, 0.28)
Alcohol use disorder models	.264	0.13 (-0.35, 0.60)	0.22 (-0.21, 0.65)	0.05 (-0.39, 0.48)	0.02 (-0.33, 0.36)
Other substance use disorder models	.400	-0.04 (-0.45, 0.37)	-0.07 (-0.45, 0.31)	0.09 (-0.29, 0.49)	-0.17 (-0.48, 0.13)
Number of DSM-5 mental disorders models		0.02 (-0.10, 0.13)	0.06 (-0.05, 0.16)	0.02 (-0.10, 0.12)	0.01 (-0.08, 0.10)
Borderline Personality Disorder models	.752	-0.21 (-0.68, 0.24)	-0.16 (-0.59, 0.28)	**0.51 (0.08, 0.96)**	-0.33 (-0.66, 0.02)
Mode of Care at Enrolment models:					
Inpatient care	.360	Reference	Reference	Reference	Reference
Outpatient care	.360	-0.26 (-0.72, 0.18)	-0.30 (-0.73, 0.16)	0.13 (-0.33, 0.58)	-0.06 (-0.43, 0.27)
Hybrid care (combination)	.280	-0.28 (-0.81, 0.22)	-0.30 (-0.77, 0.18)	-0.18 (-0.67, 0.33)	-0.12 (-0.49, 0.24)

Note: Each clinical characteristic displays the result of a separate vector autoregressive model within a dynamic structural equation model (model 1 in eMethods) with the independent variable(s) specified in the row as a between-person predictor and the NSSI variable in the columns as outcome (i.e., random effect of means) controlling for significant bivariate sociodemographic characteristics (eTable 1), including age groups (retrospective thoughts and momentary urges models) and sexual orientation (self-efficacy to resist self-injury models). Boldface for within-person associations indicates a 95% probability that the true value of the association is not null (i.e., the credibility interval does not include zero). β = Median Point Estimate, 95% CrI = 95% Credibility Interval, NSSI = Non-Suicidal Self-Injury.

© 2024 Kiekens G et al. *JAMA Network Open.*

eTable 4. Non-Suicidal Self-Injury (NSSI) Characteristics as Predictors of the Mean Intensity of NSSI Cognitions and the Occurrence of Behavior

	Proportion sample (n=125)	Mean Intensity Retrospective thoughts[a] β (95% CrL)	Mean Intensity Momentary urges[a] β (95% CrL)	Mean Intensity self-efficacy to resist NSSI[a] β (95% CrL)	Mean Propensity NSSI behavior β (95% CrL)
Lifetime NSSI Behavior models:					
11-50 times	.224	Reference	Reference	Reference	Reference
51-100 times	.200	-0.38 (-0.98, 0.15)	-0.40 (-0.94, 0.13)	0.21 (-0.38, 0.78)	-0.07 (-0.53, 0.38)
+100 times	.576	**0.50 (0.03, 0.96)**	0.42 (-0.02, 0.86)	-0.25 (-0.72, 0.25)	0.18 (-0.19, 0.55)
Lifetime Number of Methods models		0.04 (-0.05, 0.12)	0.02 (-0.06, 0.10)	-0.03 (-0.11, 0.06)	0.06 (-0.01, 0.13)
Age of onset NSSI behavior models:					
Childhood (11 years or younger)	.128	Reference	Reference	Reference	Reference
Adolescence (12-18 years)	.776	-0.10 (-0.74, 0.48)	0.01 (-0.58, 0.61)	0.29 (-0.29, 0.87)	0.21 (-0.24, 0.68)
Emerging Adulthood (19-30 years)	.096	-0.14 (-1.05, 0.72)	-0.08 (-0.90, 0.74)	0.45 (-0.36, 1.29)	0.04, (-0.63, 0.70)
Past-year NSSI thoughts models:					
5-50 times	.208	Reference	Reference	Reference	Reference
51-100 times	.224	-0.10 (-0.68, 0.43)	-0.12 (-0.65, 0.42)	-0.32 (-0.89, 0.26)	0.18 (-0.29, 0.63)
+100 times	.568	**0.54 (0.04, 1.02)**	**0.53 (0.08, 1.00)**	-0.46 (-0.94, 0.05)	0.35 (-0.03, 0.76)
Past-year NSSI behavior models:					
0-10 times	.112	Reference	Reference	Reference	Reference
11-50 times	.488	0.09 (-0.56, 0.72)	0.14 (-0.48, 0.72)	-0.39 (-1.05, 0.22)	0.41 (-0.09, 0.94)
51-100 times	.224	0.68 (-0.03, 1.39)	0.56 (-0.14, 1.24)	**-0.78 (-1.50, -0.09)**	**0.93 (0.42, 1.47)**
+100 times	.176	0.72 (-0.02, 1.45)	0.60 (-0.12, 1.30)	-0.70 (-1.42, 0.03)	**1.20 (0.68, 1.77)**
Past-month NSSI thoughts models:					
1-10 times	.232	Reference	Reference	Reference	Reference
11-30 times	.520	**0.73 (0.25, 1.16)**	**0.72 (0.29, 1.17)**	**-0.62 (-1.07, -0.16)**	**0.65 (0.29, 1.02)**
+30 times	.248	**1.13 (0.55, 1.66)**	**1.03 (0.52, 1.55)**	**-0.78 (-1.31, -0.22)**	**0.92 (0.53, 1.34)**
Past-month NSSI behavior models:					
0 times	.144	Reference	Reference	Reference	Reference
1-10 times	.616	0.21 (-0.36, 0.73)	0.16 (-0.37, 0.67)	-0.28 (-0.83, 0.27)	**0.87 (0.45, 1.37)**
+10 times	.240	0.58 (-0.10, 1.22)	0.44 (-0.19, 1.08)	-0.57 (-1.21, 0.08)	**1.56 (1.08, 2.12)**
Required Medical Treatment for NSSI models:					
No	.168	Reference	Reference	Reference	Reference
No, but it was necessary	.120	-0.16 (-0.90, 0.52)	-0.20 (-0.91, 0.48)	0.30 (-0.44, 1.02)	-0.33 (-0.96, 0.25)
Yes	.712	0.43 (-0.10, 0.92)	0.32 (-0.18, 0.82)	-0.32 (-0.81, 0.21)	-0.14 (-0.52, 0.27)
Uncontrollable urges before self-injury models					
Strongly disagree-neutral	.192	Reference	Reference	Reference	Reference
Strongly agree-agree	.808	0.36 (-0.13, 0.87)	**0.49 (0.03, 0.98)**	-0.09 (-0.57, 0.42)	0.07 (-0.31, 0.45)

Note: Each clinical characteristic displays the result of a separate vector autoregressive model within a dynamic structural equation model (model 1 in eMethods) with the independent variable(s) specified in the row as a between-person predictor and the NSSI variable in the columns as outcome (i.e., random effect of means) controlling for significant bivariate sociodemographic characteristics (eTable 1), including age groups (retrospective thoughts and momentary urges models) and sexual orientation (self-efficacy to resist self-injury models). Boldface for within-person associations indicates a 95% probability that the true value of the association is not null (i.e., the credibility interval does not include zero). β = Median Point Estimate, 95% CrI = 95% Credibility Interval, NSSI = Non-Suicidal Self-Injury.

© 2024 Kiekens G et al. *JAMA Network Open.*

eTable 5. Trends in the Course of Non-Suicidal Self-Injury across 28 Days

	Retrospective thoughts<br>B (95% CrI)	Momentary urges<br>B (95% CrI)	Self-efficacy to resist NSSI<br>B (95% CrI)	NSSI behavior<br>B (95% CrI)
Linear trend models				
day linear	0.000 (-0.005, 0.006)	0.001 (-0.005, 0.007)	**0.009 (0.002, 0.018)**	**-0.102 (-0.140, -0.065)**
Quadratic trend models				
day linear	0.001 (-0.006, 0.007)	0.001 (-0.005, 0.007)	**0.009 (0.001, 0.018)**	**-0.103 (-0.140, -0.067)**
day quadratic	-0.003 (-0.009, 0.004)	0.000 (-0.007, 0.006)	0.003 (-0.006, 0.011)	-0.022 (-0.065, 0.021)
Cubic trend models				
day linear	-0.004 (-0.019, 0.011)	-0.004 (-0.020, 0.013)	**0.023 (0.006, 0.042)**	**-0.111 (-0.204, -0.019)**
day quadratic	-0.004 (-0.010, 0.003)	-0.001 (-0.007, 0.006)	0.005 (-0.003, 0.013)	-0.022 (-0.062, 0.020)
day cubic	-0.003 (-0.005, 0.011)	0.003 (-0.006, 0.011)	-0.008 (-0.017, 0.001)	0.005 (-0.044, 0.052)

Note: Within-person unstandardized lagged associations based on regular surveys with two-hour intervals estimated within multilevel vector autoregressive models in dynamic structural equation framework (model 2 in eMethods). Each polynomial function displays the result of a separate vector autoregressive model with the dependent variable specified in the column as the outcome. Boldface indicates a 95% probability that the true value of the association is not null (i.e., the credibility interval does not include zero). B = Median Point Estimate, 95% CrI = 95% Credibility Interval, NSSI = Non-Suicidal Self-Injury.

© 2024 Kiekens G et al. *JAMA Network Open.*

eTable 6. Daily Variation in the Course of Non-Suicidal Self-Injury

	Retrospective thoughts B (95% CrI)	Momentary urges B (95% CrI)	Self-efficacy to resist NSSI B (95% CrI)	NSSI behavior B (95% CrI)
One-day (every 24 hours) cycle models				
cosT	-0.005 (-0.017, 0.006)	-0.008 (-0.019, 0.004)[a]	0.006 (-0.007, 0.020)	0.031 (-0.039, 0.098)
sinT	-0.011 (-0.029, 0.007)	-0.005 (-0.023, 0.013)[a]	-0.006 (-0.030, 0.017)	**-0.355 (-0.469, -0.237)**
Two-day (every 12 hours) cycle models				
cos2T	0.001 (-0.008, 0.009)	-0.001 (-0.010, 0.008)[a]	0.008 (-0.002, 0.019)	**0.183 (0.125, 0.242)**
sin2T	-0.005 (-0.013, 0.004)	-0.006 (-0.014, 0.003)[a]	0.005 (-0.005, 0.016)	-0.035 (-0.091, 0.023)
Categorical survey models:				
morning assessment (10-11:59 am)	Reference	Reference	Reference	Reference
noon assessment (12-1:59 pm)	**-0.032 (-0.054, -0.011)**	-0.003 (-0.026, 0.018)	0.018 (-0.010, 0.045)	**-0.488 (-0.633, -0.337)**
early afternoon assessment (2-3:59 pm)	**-0.024 (-0.045, -0.002)**	-0.004 (-0.026, 0.019)	0.010 (-0.016, 0.036)	**-0.486 (-0.624, -0.338)**
late afternoon assessment (4-5:59 pm)	**-0.025 (-0.045, -0.004)**	-0.003 (-0.026, 0.019)	-0.005 (-0.031, 0.022)	**-0.607 (-0.759, -0.471)**
early evening assessment (6-7:59 pm)	-0.016 (-0.037, 0.005)	0.005 (-0.016, 0.028)	-0.001 (-0.028, 0.025)	**-0.409 (-0.545, -0.266)**
late evening assessment (8-9:59 pm)	-0.020 (-0.043, 0.001)	0.008 (-0.013, 0.031)	0.000 (-0.027, -0.025)	**-0.433 (-0.566, -0.292)**
Continuous survey models:	-0.001 (-0.005, 0.002)	0.002 (-0.001, 0.006)	-0.002 (-0.006, 0.002)	**-0.065 (-0.088, -0.043)**

Note: Within-person unstandardized lagged associations based on regular surveys with two-hour intervals estimated within multilevel vector autoregressive models in dynamic structural equation framework (model 2 in eMethods). One-day and two-day cycles and survey models display the result of a separate vector autoregressive model with the dependent variable specified in the column as the outcome. [a] Covariances of random effects with residual variances had to be excluded for the model to converge. Linear trends were included for self-efficacy to resist NSSI and NSSI behavior in these models. Boldface indicates a 95% probability that the true value of the association is not null (i.e., the credibility interval does not include zero). B = Median Point Estimate, 95% CrI = 95% Credibility Interval, NSSI = Non-Suicidal Self-Injury

© 2024 Kiekens G et al. *JAMA Network Open.*

eTable 7. Within-day Trajectories of NSSI behavior using Timing of Event Marker

	NSSI behavior registered with event marker B (95% CrI)
One-day (every 24 hours) cycle model	
cosT	-0.013 (-0.079, 0.052)
sinT	**-0.293 (-0.378, -0.220)**
within-level R^2 averaged across individuals	0.136 (0.107, 0.170)
Two-day (every 12 hours) cycle model	
cos2T	0.063 (-0.006, 0.132)
sin2T	**-0.196 (-0.273, -0.127)**
within-level R^2 averaged across individuals	0.128 (0.099, 0.165)
Linear within-day trajectory model:	
time linear	**0.225 (0.173, 0.283)**
within-level R^2 averaged across individuals	0.145 (0.116, 0.178)
Quadratic within-day trajectory model:	
time linear	**0.238 (0.180, 0.304)**
time quadratic	-0.034, (-0.093, 0.022)
within-level R^2 averaged across individuals	0.154 (0.121 0.194)
Cubic within-day trajectory modelv:	
time linear	**0.361 (0.226, 0.504)**
time quadratic	-0.009 (-0.068, 0.059)
time cubic	**-0.068 (-0.140, -0.002)**
within-level R^2 averaged across individuals	0.152 (0.121, 0.186)
Quartic within-day trajectory model:	
time linear	**0.474 (0.317, 0.673)**
time quadratic	**-0.319 (-0.528, -0.104)**
time cubic	**-0.118 (-0.200, -0.045)**
time quartic	**0.124 (0.045, 0.204)**
within-level R^2 averaged across individuals	0.171 (0.133, 0.216)
Quintic within-day trajectory model:	
time linear	0.114 (-0.101, 0.346)
time quadratic	**-0.549 (-0.896 -0.304)**
time cubic	**0.546 (0.228, 0.907)**
time quartic	**0.200 (0.110, 0.320)**
time quintic	**-0.204 (-0.319, -0.105)**
within-level R^2 averaged across individuals	0.213 (0.163, 0.284)

Note: Within-person unstandardized lagged associations based on regular surveys with one-hour intervals estimated within multilevel vector autoregressive models in dynamic structural equation framework (model 2 in eMethods). One-day and two-day cycles and polynomial models display the result of a separate vector autoregressive model with the dependent variable specified in the column as the outcome. Linear trends were included for self-efficacy to resist NSSI and NSSI behavior in these models. Boldface indicates a 95% probability that the true value of the association is not null (i.e., the credibility interval does not include zero). B = Median Point Estimate, 95% CrI = 95% Credibility Interval, NSSI = Non-Suicidal Self-Injury.

eTable 8. Weekly Variation in the Course of Non-Suicidal Self-Injury

	Retrospective thoughts<br>B (95% CrI)	Momentary urges<br>B (95% CrI)	Self-efficacy to resist NSSI<br>B (95% CrI)	NSSI behavior<br>B (95% CrI)
Day-of-week models with reference all other days:				
Monday	**0.021 (0.004, 0.038)**	0.014 (-0.003, 0.030)	-0.019 (-0.041, 0.002)	0.077 (-0.024, 0.179)
Tuesday	0.010 (-0.007, 0.027)	0.005 (-0.012, 0.021)	-0.010 (-0.031, 0.010)	0.099 (-0.001, 0.194)
Wednesday	0.000 (-0.017, 0.017)	0.003 (-0.014, 0.020)	-0.003 (-0.024, 0.019)	-0.048 (-0.151, 0.052)
Thursday	0.002 (-0.015, 0.019)	-0.001 (-0.019, 0.017)	-0.009 (-0.031, 0.012)	-0.032 (-0.132, 0.066)
Friday	-0.006 (-0.022, 0.010)	-0.006 (-0.023, 0.010)	-0.001 (-0.022, 0.019)	-0.015 (-0.115, 0.086)
Saturday	**-0.026 (-0.044, -0.009)**	-0.012 (-0.030, 0.006)	**0.045 (0.023, 0.070)**	**-0.136 (-0.244, -0.027)**
Sunday	-0.001 (-0.018, 0.015)	-0.002 (-0.018, 0.015)	0.002 (-0.018, 0.023)	0.029 (-0.069, 0.131)
Day-of-week models with reference start of the week:				
Monday	*Reference*	*Reference*	*Reference*	*Reference*
Tuesday	-0.009 (-0.032, 0.011)	-0.009 (-0.030, 0.013)	0.008 (-0.020, 0.035)	0.010 (-0.120, 0.134)
Wednesday	-0.018 (-0.041, 0.004)	-0.010 (-0.032, 0.012)	0.014 (-0.014, 0.044)	-0.108 (-0.237, 0.025)
Thursday	-0.017 (-0.039, 0.005)	-0.013 (-0.037, 0.009)	0.008 (-0.021, 0.036)	-0.100 (-0.231, 0.030)
Friday	**-0.023 (-0.046, -0.002)**	-0.018 (-0.039, 0.003)	0.015 (-0.012, 0.044)	-0.081 (-0.211, 0.040)
Saturday	**-0.041 (-0.063, -0.019)**	**-0.023 (-0.045, -0.001)**	**0.058 (0.030, 0.090)**	**-0.188 (-0.322, -0.062)**
Sunday	-0.019 (-0.041, 0.003)	-0.014 (-0.036, 0.008)	0.020 (-0.006, 0.048)	-0.047 (-0.180, 0.086)
Weekend-versus-week (reference) models	**-0.017 (-0.029, -0.005)**	-0.008 (-0.021, 0.004)	**0.024 (0.008, 0.041)**	-0.067 (-0.144, 0.010)

Note: Within-person unstandardized lagged associations based on regular surveys with two-hour intervals estimated within multilevel vector autoregressive models in dynamic structural equation framework (model 2 in eMethods). Day-of-week and weekend (from Friday 5 pm till Sunday evening) versus week models display the result of a separate vector autoregressive model with the dependent variable specified in the column as the outcome. Linear trends were included for self-efficacy to resist NSSI and NSSI behavior in these models. Boldface indicates a 95% probability that the true value of the association is not null (i.e., the credibility interval does not include zero). B = Median Point Estimate, 95% CrI = 95% Credibility Interval, NSSI = Non-Suicidal Self-Injury

© 2024 Kiekens G et al. *JAMA Network Open.*

eTable 9. Age-Specific Moderation of the Temporal Associations Between Non-Suicidal Self-Injury Cognitions and Behavior

	**Prediction retrospective thoughts in full multivariable regression**[a] (n=124, i=14,984 longitudinal assessments)		
Lagged predictors:	**Intercept** **B (95% CrI)**	**Slope emerging adults** **B (95% CrI)**	**Slope adults** **B (95% CrI)**
Retrospective thoughts$_{T-2h}$	0.06 (-0.03, 0.15)	-0.04 (-0.15, 0.06)	-0.05 (-0.20, 0.10)
Momentary urges$_{T-2h}$	**0.35 (0.18, 0.52)**	0.09 (-0.11, 0.28)	-0.04 (-0.31, 0.24)
Self-efficacy to resist NSSI$_{T-2h}$	**-0.12 (-0.20, -0.04)**	-0.02 (-0.10, 0.07)	-0.11 (-0.23, 0.02)
NSSI behavior since last assessment$_{T-2h}$	0.06 (-0.11, 0.21)	-0.12 (-0.31, 0.07)	-0.16 (-0.42, 0.11)
	**Prediction momentary urges in full multivariable regression**[a] (n=124, i=14,947 longitudinal assessments)		
Lagged predictors:	**Intercept** B (95% CrI)	**Slope emerging adults** B (95% CrI)	**Slope adults** B (95% CrI)
Retrospective thoughts$_{T-2h}$	**0.31 (0.19, 0.42)**	0.02 (-0.12, 0.15)	-0.07 (-0.26, 0.12)
Momentary urges$_{T-2h}$	0.03 (-0.05, 0.11)	-0.02 (-0.11, 0.08)	0.01 (-0.11, 0.14)
Self-efficacy to resist NSSI$_{T-2h}$	**-0.20 (-0.28, -0.12)**	0.01 (-0.08, 0.10)	**-0.14 (-0.27, -0.01)**
NSSI behavior since last assessment$_{T-2h}$	-0.08 (-0.22, 0.07)	-0.06 (-0.23, 0.11)	-0.11 (-0.34, 0.12)
	**Prediction self-efficacy to resist NSSI in full multivariable regression**[a] (n=123, i=14,853 longitudinal assessments)		
Lagged predictors:	**Intercept** **B (95% CrI)**	**Slope emerging adults** **B (95% CrI)**	**Slope adults** **B (95% CrI)**
Retrospective thoughts$_{T-2h}$	**-0.09 (-0.17, -0.01)**	0.01 (-0.09, 0.11)	0.00 (-0.14, 0.14)
Momentary urges$_{T-2h}$	**-0.14 (-0.22, 0.06)**	-0.05 (-0.13, 0.04)	-0.05 (-0.18, 0.08)
Self-efficacy to resist NSSI$_{T-2h}$	**0.16 (0.07, 0.26)**	-0.04 (-0.15, 0.08)	0.10 (-0.06, 0.26)
NSSI behavior since last assessment$_{T-2h}$	0.04 (-0.10, 0.19)	0.07 (-0.09, 0.24)	0.13 (-0.11, 0.36)
	**Prediction NSSI behavior next 2 hours in full multivariable regression**[a] (n=105, i=12,771 longitudinal assessments)		
Lagged predictors:	**Intercept** **B (95% CrI)**	**Slope emerging adults** **B (95% CrI)**	**Slope adults** **B (95% CrI)**
Retrospective thoughts$_{T-2h}$	**0.21 (0.08, 0.35)**	-0.03 (-0.21, 14)	0.00 (-0.23, 0.21)
Momentary urges$_{T-2h}$	-0.01 (-0.16, 0.15)	0.09 (-0.11, 0.28)	0.16 (-0.09, 0.40)
Self-efficacy to resist NSSI$_{T-2h}$	**-0.16 (-0.30, -0.02)**	0.03 (-0.14, 0.21)	0.12 (-0.09, 0.33)
NSSI behavior since last assessment$_{T-2h}$	0.15 (-0.02, 0.32)	-0.01 (-0.21, 0.18)	-0.20 (-0.45, 0.07)

Note: [a] Within-person unstandardized lagged associations based on regular surveys with two-hour intervals estimated within multilevel vector autoregressive models in a dynamic structural equation framework (model 6 in eMethods). Each of the four models includes all the lagged variable(s) mentioned in the rows jointly as within-predictors for the outcome under investigation, with age groups as between-person predictors of the random effects of the autoregressive and crossregressive associations over time (adolescents are the reference group). Linear trends were included for self-efficacy to resist NSSI and NSSI behavior in these models. Boldface indicates a 95% probability that the true value of the association is not null (i.e., the credibility interval does not include zero). B = Median Point Estimate, 95% CrI = 95% Credibility Interval, NSSI = Non-Suicidal Self-Injury.

© 2024 Kiekens G et al. *JAMA Network Open.*

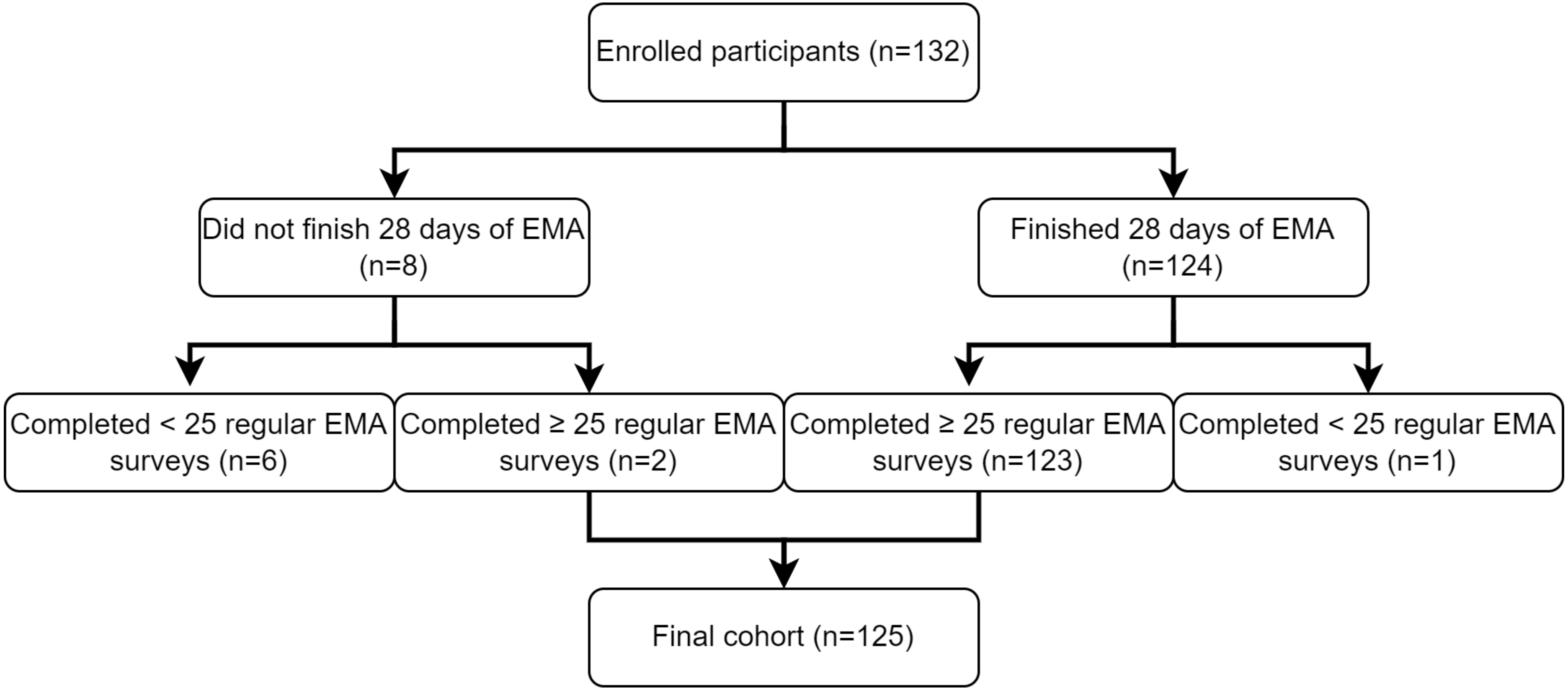

eFigure 7. Flowchart of Analytical Sample

© 2024 Kiekens G et al. *JAMA Network Open.*

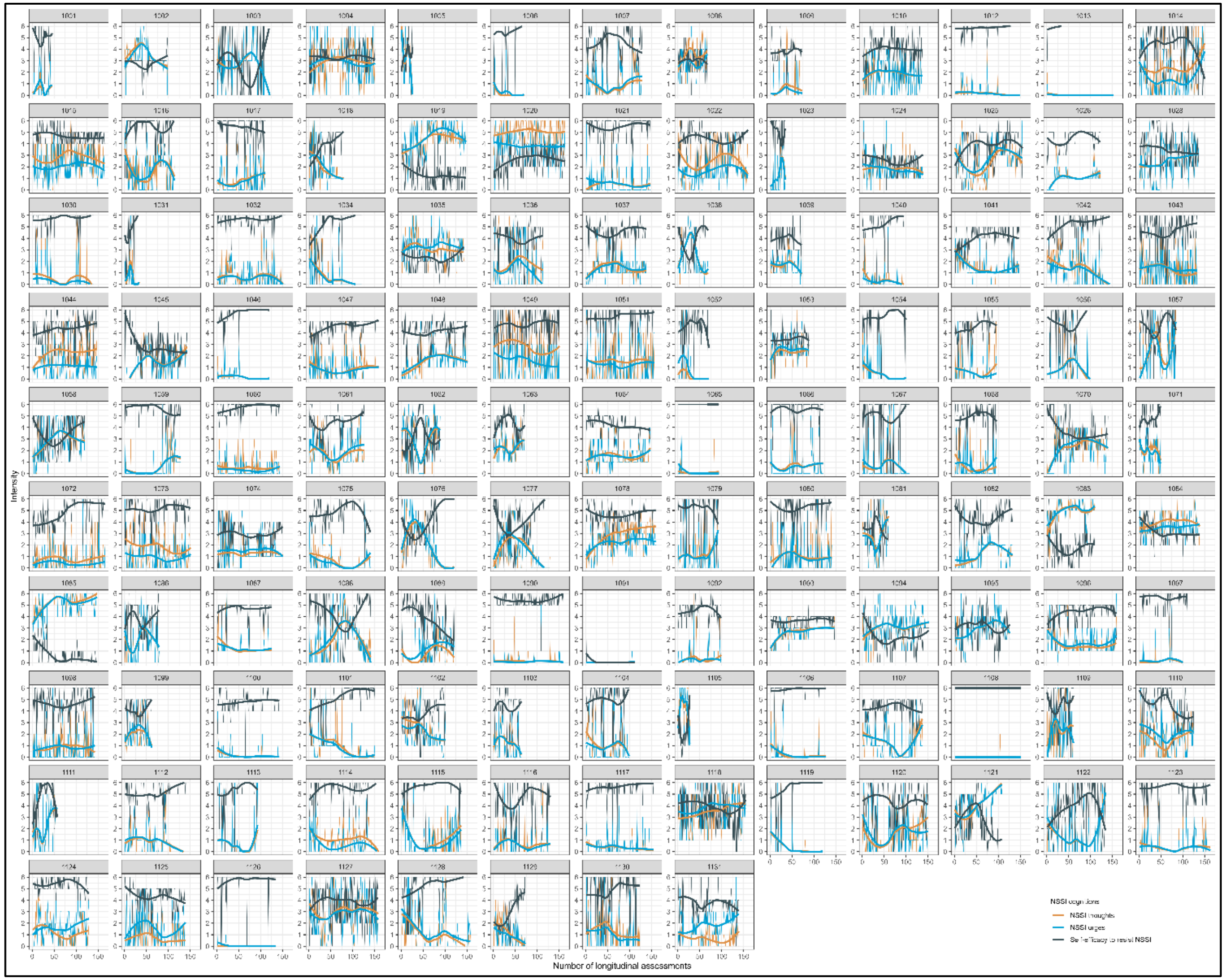

eFigure 8. One-Hundred Twenty-Five Individual Time Series Plots with Smoothed Trend Lines for Non-Suicidal Self-Injury (NSSI) Cognitions

© 2024 Kiekens G et al. *JAMA Network Open.*

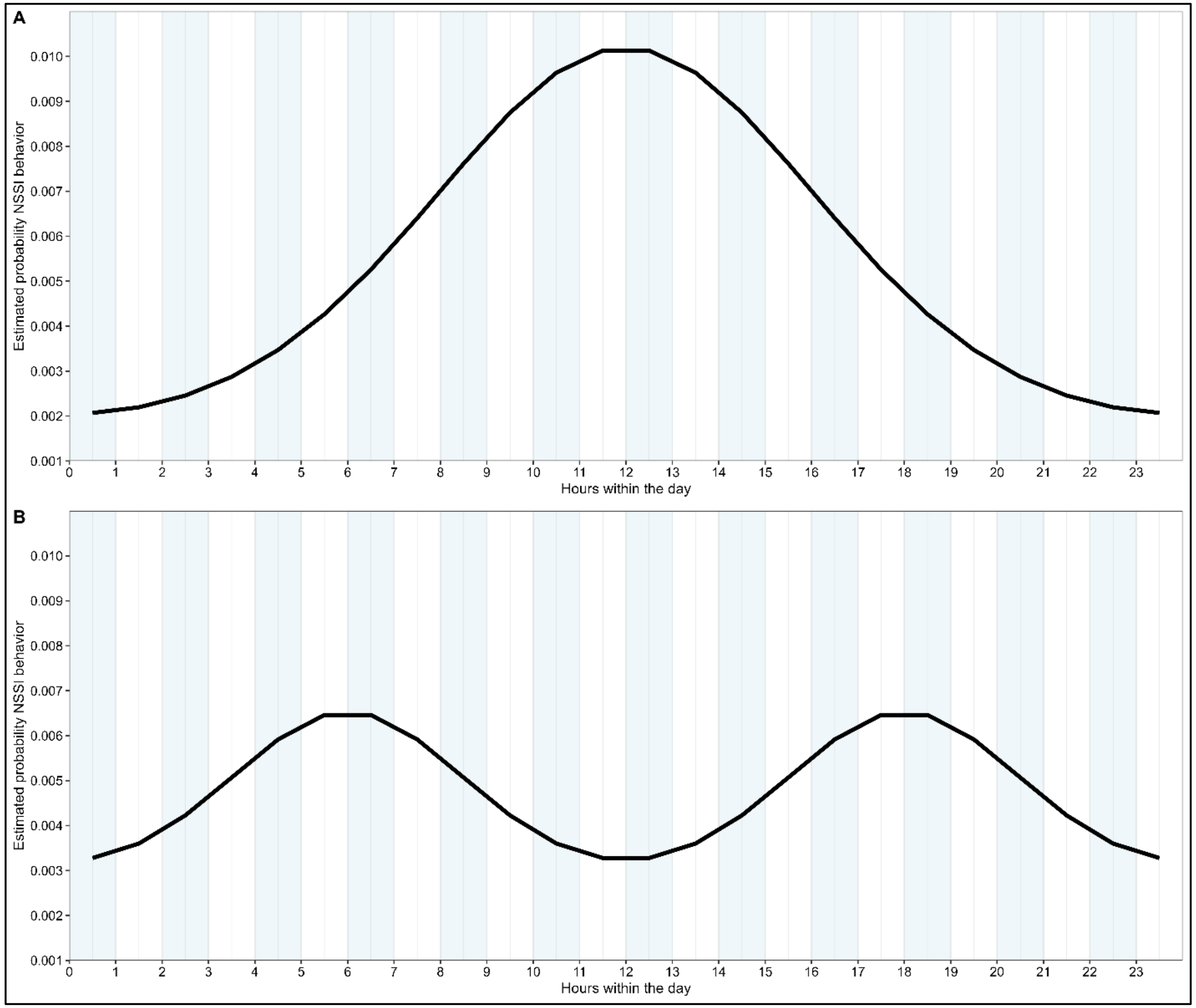

eFigure 9. Daily Cycles for Non-Suicidal Self-Injury Behavior using Timing of Event Marker

Note: (A) One-day (every 24 hours) cycle, (B) Two-day (every 12 hours) cycles. Probability is based on estimate after two weeks of monitoring (i.e., the middle of the study when linear trend equals 0; eTable 6).

© 2024 Kiekens G et al. *JAMA Network Open.*
