# Supplementary material for: The Short-Term Course of Nonsuicidal Self-Injury Among Individuals Seeking Psychiatric Treatment
Source: JAMA Netw Open. 2024 Oct 22;7(10):e2440510. doi: 10.1001/jamanetworkopen.2024.40510 (PMC11581677; doi:10.1001/jamanetworkopen.2024.40510)
Supplement: Supplement 2. — Data Sharing Statement [file jamanetwopen-e2440510-s002.pdf]

Data Sharing Statement

Kiekens. The Short-Term Course of Nonsuicidal Self-Injury Among Individuals Seeking Psychiatric Treatment. *JAMA Netw Open*. Published October 22, 2024.
doi:10.1001/jamanetworkopen.2024.40510

Data
Data available: No

Additional Information
Explanation for why data not available: The datasets generated for this study are available upon reasonable request to the corresponding author and after signing a data-sharing agreement.
